# MAP2 caps tau fibrils and inhibits aggregation

**DOI:** 10.1016/j.jbc.2023.104891

**Published:** 2023-06-05

**Authors:** Michael R. Holden, Brad J. Krzesinski, Hilary A. Weismiller, Justin R. Shady, Martin Margittai

**Affiliations:** Department of Chemistry and Biochemistry, University of Denver, Denver, Colorado, USA

**Keywords:** aggregation, Alzheimer’s disease, amyloid, fibril, inhibition, MAP2, prion, seeding, tauopathy, tau protein

## Abstract

Fibrils of the microtubule-associated protein tau are intimately linked to the pathology of Alzheimer’s disease (AD) and related neurodegenerative disorders. A current paradigm for pathology spreading in the human brain is that short tau fibrils transfer between neurons and then recruit naive tau monomers onto their tips, perpetuating the fibrillar conformation with high fidelity and speed. Although it is known that the propagation could be modulated in a cell-specific manner and thereby contribute to phenotypic diversity, there is still limited understanding of how select molecules are involved in this process. MAP2 is a neuronal protein that shares significant sequence homology with the repeat-bearing amyloid core region of tau. There is discrepancy about MAP2’s involvement in pathology and its relationship with tau fibrillization. Here, we employed the entire repeat regions of 3R and 4R MAP2, to investigate their modulatory role in tau fibrillization. We find that both proteins block the spontaneous and seeded aggregation of 4R tau, with 4R MAP2 being slightly more potent. The inhibition of tau seeding is observed *in vitro*, in HEK293 cells, and in AD brain extracts, underscoring its broader scope. MAP2 monomers specifically bind to the end of tau fibrils, preventing recruitment of further tau and MAP2 monomers onto the fibril tip. The findings uncover a new function for MAP2 as a tau fibril cap that could play a significant role in modulating tau propagation in disease and may hold promise as a potential intrinsic protein inhibitor.

Neurofibrillary tangles composed of straight and paired helical filaments are a key pathological hallmark of Alzheimer’s disease (AD) (1, 2, 3). The filaments are concentrated in the soma of neurons and are made of the microtubule-associated protein tau. Over the past several years, it has become increasingly evident that short fibrils and oligomers of tau can transfer from one neuron to another and thereby spread pathology throughout the brain in a prion-like manner (4, 5, 6). Apart from AD, there are many other neurodegenerative disorders that are characterized by filamentous tau inclusions (7, 8). Collectively, these disorders are referred to as tauopathies. The molecular mechanisms of tau pathology spreading are likely very similar in different disorders, although the brain regions that are affected vary widely. A unifying theme of tau fibril architecture is the cross-β structure in which β-strands from neighboring tau molecules are stacked parallel and in-register, forming multiple extended β-sheets along the long fibril axis (9) that intricately pack against each other. In AD, tau filaments assume a cross-β/β-helical fold (10). In other tauopathies, the tau filament folds are different, based on variations in core size and differences in packing (11).

In the adult human brain, six different tau isoforms are expressed that range in size from 352 to 441 amino acids and vary by the presence of zero, one, or two inserts at the N terminus, and the inclusion or not of the second of four microtubule-binding repeats in the C-terminal half of the protein. The latter distinction allows categorization into two groups of tau isoforms, one having three repeats (3R tau) and the other one having four repeats (4R tau). Both types of isoforms occur in similar abundance, although within each group there are differences in expression (12, 13). The repeat region is a major contributor to the core structure of tau fibrils (14, 15). In AD, and a few other disorders such as primary age-related tauopathy and chronic traumatic encephalopathy, tau filaments are composed of all isoforms (16, 17, 18, 19). In other tauopathies, there is preferential deposition of either 3R tau (20) or 4R tau (21, 22, 23). It is clear that these differences have a direct effect on the fibril structure. However, even fibrils that are made of only 4R tau can assume different conformations as exemplified by the distinct structures of tau fibrils found in corticobasal degeneration and progressive supranuclear palsy (11, 24), both of which are 4R tauopathies. The factors determining the particular fold of a tau fibril are not fully understood, although the incorporation of select cofactors (25, 26), differences in tau posttranslational modifications (27), and interactions with various modulating proteins could play a role.

Tau belongs to a family of microtubule-associated proteins including MAP2, a protein that is also prevalently expressed in neurons (28). Like tau, MAP2 features different splice variants that may have either three or four microtubule binding repeats (29), with 3R isoforms being predominant. *In vitro* experiments have demonstrated that MAP2 forms either fibrillar (30, 31) or granular, non-amyloidogenic aggregates (32, 33). Despite its similarities with tau, there is no evidence, however, that MAP2 forms fibrillar deposits in the human AD brain. Indeed, straight and paired helical filaments are almost exclusively made of tau (10). Nevertheless, small quantities of MAP2 have been identified in neurofibrillary tangles (34, 35), and interactome studies in human AD brain have captured MAP2 together with oligomeric tau (35, 36). Furthermore, MAP2 was found sequestered in a sedimentable fraction from AD brain homogenate containing small tau aggregates/oligomers referred to as AD P-tau (37). Markedly, in this sequestered form, MAP2 lost its ability to facilitate microtubule assembly (37). Combined, these studies suggest that there must be some interaction between aggregated tau and MAP2. However, the nature of this interaction and the potential implications for tau aggregation are completely unknown.

Here, we set out to investigate the interactions between tau and MAP2 and to illuminate their impact on tau fibrillization. The study demonstrates that MAP2 blocks the spontaneous and seeded aggregation of tau, and that it caps the end of tau fibrils, offering a new potent mechanism of inhibition.

## Results

### MAP2 inhibits spontaneous tau aggregation

**Figure 1:**
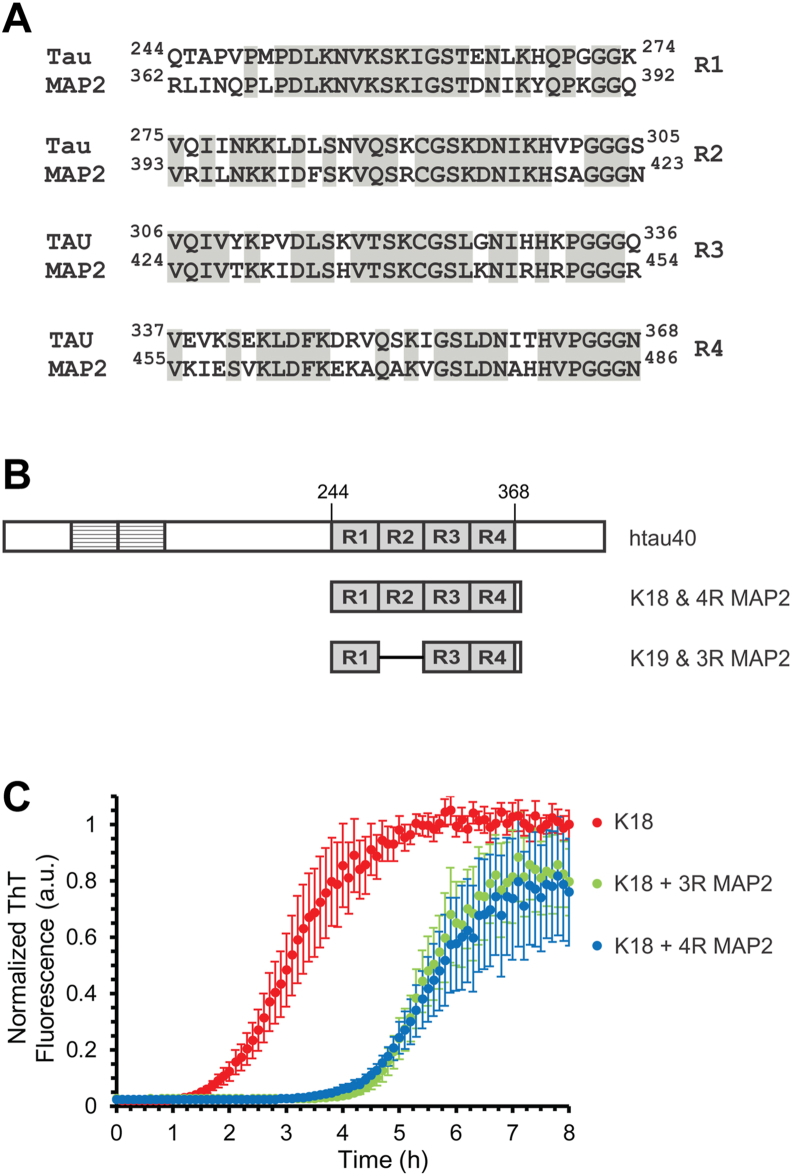
**MAP2 inhibits spontaneous tau aggregation.***A*, amino acid sequence alignment of the microtubule-binding repeats (R1-R4) of tau and MAP2 reveals 68% sequence identity (*shaded*) and 85% similarity. Depending on the isoform, R2 may be present or not. Numbering is based on the largest isoform of tau, htau40 (Uniprot: P10636-8), and a four-repeat variant of MAP2, isoform 4, also referred to as MAP2d (Uniprot: P11137-4). *B*, schematic diagram of truncated tau (K18 and K19) and MAP2 (3R MAP2 and 4R MAP2) in relation to htau40 (the largest tau isoform). *C*, ThT fluorescence measurements of 25 μM K18, aggregating in the absence (*red trace*) or presence of 3.2 μM MAP2 (3R MAP2, *green trace*; 4R MAP2, *blue trace*) in triplicate at 37 °C. The t_1/2_ values for these reactions were 3.0 and 5.4 h, respectively. Error bars represent means ± SD.

Tau and MAP2 share significant sequence similarity in the microtubule binding repeats (Fig. 1*A*), the same region that forms part of the core region of pathological tau filaments (15). In the first set of experiments, we sought to determine whether MAP2 can modulate spontaneous tau aggregation. As a model system for tau aggregation, we chose K18, a four-repeat version (amino acids 244–372), that spans the entire repeat region plus four additional residues at the C terminus (Fig. 1*B*). This protein aggregates significantly faster than K19, the corresponding three-repeat version (38, 39) (Fig. 1*B*). We generated MAP2 variants that match the sizes of the K18 and K19 constructs. Henceforth, these variants are referred to as 4R MAP2 and 3R MAP2, respectively (Fig. 1*B*). To monitor tau aggregation, 25 μM K18 were incubated with a twofold molar excess of heparin and 20 μM of Thioflavin T (ThT) while intermittently agitating (see Experimental procedures). An increase in ThT fluorescence signals fibril formation as the dye binds to the newly formed β-sheet structure (40). The ThT trace for K18 aggregation (Fig. 1*C*) shows the typical characteristics of a nucleation-dependent process: a lag phase, an elongation phase, and a saturation phase. Half-maximal aggregation, which is reflected in t_1/2_, was reached after 3.0 h. Notably, the addition of substoichiometric quantities of 3R MAP2 or 4R MAP2 (3.2 μM) at the beginning of the reaction increased t_1/2_ by almost twofold (Fig. 1*C*). These results suggest that MAP2 acts as an inhibitor of spontaneous tau aggregation.

### MAP2 inhibits seeded tau aggregation

**Figure 2:**
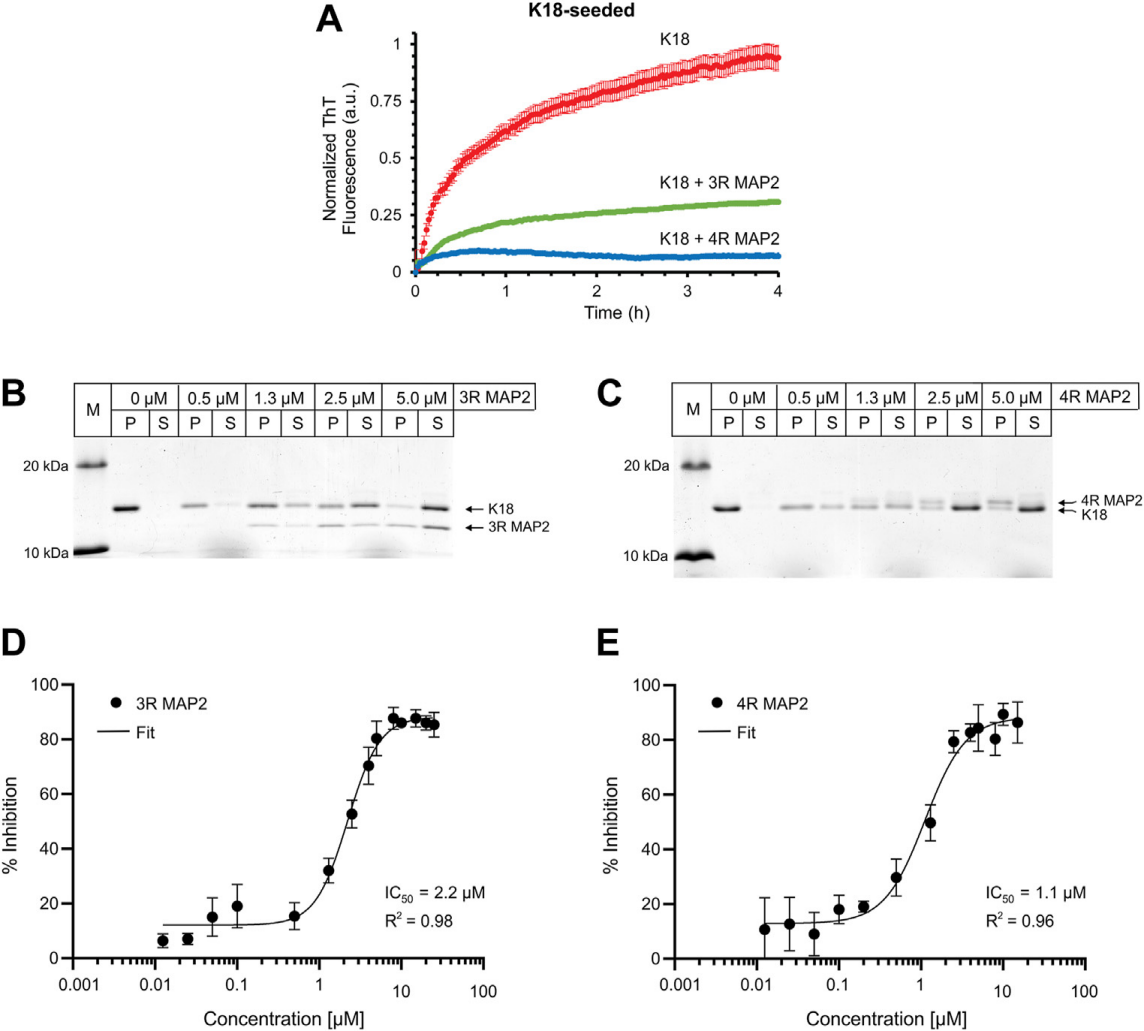
**MAP2 inhibits seeded tau aggregation.***A*, 10 μM K18 monomers were mixed with 3% seeds (monomer equivalents) either in the absence or in the presence of 2.5 μM MAP2 (3R and 4R). Fibril growth was monitored by ThT fluorescence for 4 h at 37 °C. –MAP2, red trace; +3R MAP2, green trace; +4R MAP2, blue trace. *B* and *C*, coomassie-stained SDS-PAGE gels of 3R and 4R MAP2 inhibiting K18-seeded tau aggregation, respectively. In these experiments, 10 μM K18 monomers were mixed with 10% seeds (monomer equivalents) and incubated for 6 h at 37 °C in the presence or absence of MAP2. The samples were then sedimented for 30 min at 130,000*g* and equivalent volumes were loaded onto the gels. The sedimentation experiments were repeated in triplicate using a broad range of MAP2 concentrations (Figs. S1 and S2). Band intensities were calculated using the equation I_P_/(I_P_ + I_S_), where I_P_ and I_S_ are the integrated band intensities measured in ImageJ from the Coomassie-stained gels for the pellets (I_P_) and supernatants (I_S_). Percentage of inhibition is plotted *versus* the concentration of 3R MAP2 (*D*) and 4R MAP2 (*E*). IC_50_ values were computed by fitting the curves with a four-parameter logistic fit function in GraphPad Prism. Error bars represent means ± SD. M, protein marker; P, pellet; S, supernatant.

To test whether MAP2 interferes with fibril elongation, a tau seeding assay was employed. For this purpose, K18 fibrils were first sheared to produce small seeds and then added to K18 monomers. Fibril elongation was monitored by ThT fluorescence. In the absence of MAP2, K18 was effectively recruited onto the fibril as judged by the steady increase in ThT fluorescence (Fig. 2*A*, red trace). In the presence of substoichiometric quantities of 3R or 4R MAP2, the ThT signals were greatly reduced (Fig. 2*A*, green and blue traces, respectively) suggesting diminished recruitment of K18 monomers. To further quantify the inhibitory effects of MAP2 on tau seeding, a separate set of experiments was performed in which the insoluble material was sedimented after incubation and then analyzed by SDS-PAGE and Coomassie staining. In the absence of MAP2, K18 was found in the pellet (Fig. 2, *B* and *C*), confirming efficient recruitment of K18 monomers onto the seeds. In the presence of increasing concentrations of 3R (Fig. 2*B*) and 4R (Fig. 2*C*) MAP2, K18 shifted from pellet to supernatant, suggesting that recruitment of tau monomers onto tau seeds was perturbed. To determine the concentrations at which tau seeding was half maximally inhibited (IC_50_), the sedimentation experiments were expanded to encompass a broader range of MAP2 concentrations (Figs. S1 and S2). Analyses of these data revealed IC_50_ values of 2.2 μM for 3R MAP2 (Fig. 2*D*) and 1.1 μM for 4R MAP2 (Fig. 2*E*).

It was noticed that both 3R MAP2 and 4R MAP2 partitioned in the pellet fractions after inhibition of K18-seeded growth (Figs. 2, *B* and *C*; S1 and S2), indicating that the proteins must have aggregated during incubation. A molecular scenario in which aggregation was due to MAP2 elongating onto K18 seeds appeared unlikely because of the lack of ThT fluorescence (Fig. 2*A*). This conclusion was further supported by the observation of mostly amorphous aggregates when 3R and 4R MAP2 were incubated in the presence of heparin (Fig. S3). Together, these findings indicate that the MAP2 variants combine in a non-amyloidogenic fashion. To examine whether MAP2 may have inhibited tau elongation by depleting the pool of heparin, we next repeated the seeding experiments using increasing concentrations of heparin. Raising the molar excess of heparin over MAP2 from 4- to 16-fold did not have any effect on the seeded aggregation of tau (Fig. S4) suggesting that heparin depletion is not a major mechanism of inhibition. To better understand what species of MAP2 interfere with tau elongation, we incubated MAP2 for 16 h in the presence or absence of heparin and then added the reactions to the seeding assay. Notably, amorphous MAP2 aggregates that formed after heparin addition did not inhibit tau aggregation, MAP2 incubated without heparin, however, did (Fig. S5). These results suggest that 3R and 4R MAP2 monomers are the main species inhibiting tau elongation.

### MAP2 associates with tau fibrils

**Figure 3:**
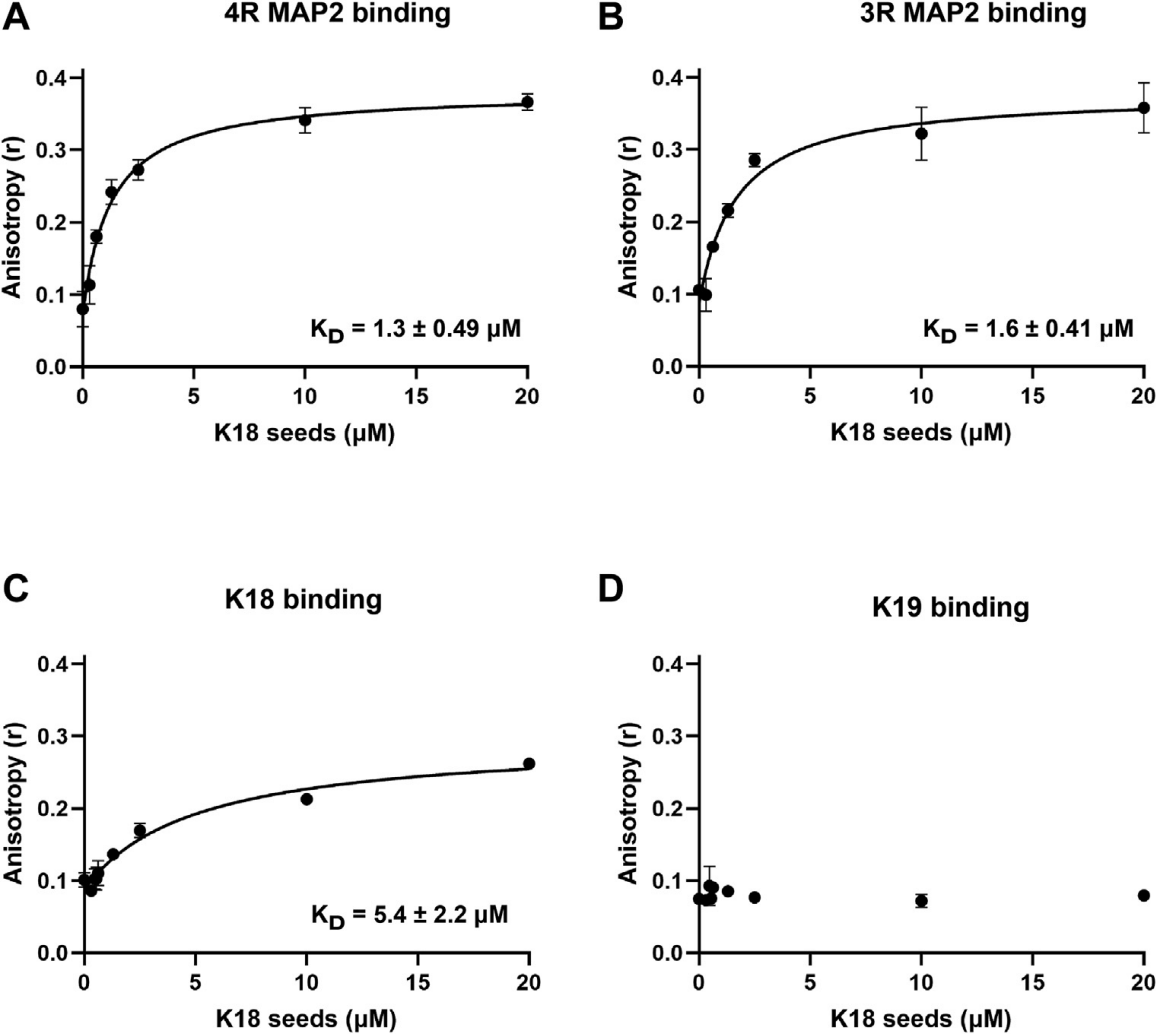
**MAP2 interacts with tau fibrils.** Increasing concentrations of K18 seeds (0–20 μM monomer equivalents) were added to 100 nM Atto 633-labeled protein monomers and equilibrated at 22 °C. Binding was measured by fluorescence anisotropy (r). The reactions included following monomers: (*A*) 4R MAP2; (*B*) 3R MAP2; (*C*) K18; (*D*) K19. All measurements were performed in triplicate (independent replicates). Error bars represent means ± SD. The data, except for those of K19 binding, were fit with a one-site binding model (see Experimental procedures). The apparent K_D_ values are provided in the graphs.

To determine whether MAP2 monomers physically interact with K18 fibrils, we utilized fluorescence anisotropy, a technique that is sensitive to changes in rotational correlation times (41). Such changes are expected upon protein binding. K19 monomers served as a control, since previous findings indicated that this protein is not incorporated into the fibril (42, 43). In a first step, the fluorophore, Atto633-maleimide, was linked to a single cysteine in K18 (position 322) and equivalent cysteines in K19, 3R MAP2, and 4R MAP2 (Fig. 1). The position was chosen because it resides in the structured core of tau fibrils and transitions from high to low mobility when intrinsically disordered tau converts into the fibrillar state (44). The labeled proteins (100 nM) were titrated with increasing concentrations of seeds (0–20 μM monomer equivalents) followed by anisotropy measurements. The hyperbolic curves observed for labeled 4R MAP2, 3R MAP2, and K18 (Fig. 3, *A*–*C*) suggest that all three of these proteins are bound to seeds. Notably, no changes in anisotropy were detected for labeled K19 (Fig. 3*D*) in agreement with the previously observed seeding barrier between the two proteins (42). The apparent dissociation constants suggest that 4R MAP2 (K_D_ = 1.3 μM) and 3R MAP2 (K_D_ = 1.6 μM) monomers have a higher affinity for K18 seeds than K18 (K_D_ = 5.4 μM) monomers have. The values are in good agreement with the IC_50_ values (Fig. 2, *D* and *E*) corroborating that the inhibition of K18 aggregation is due to binding. It is important to note that the dissociation constants are based on monomer equivalents, not seed concentrations. Given that tip-sonicated K18 seeds have an average length of ∼64 nm (45) and that the spacing between tau monomers in the fibrils is 0.47 nm, a typical seed contains ∼136 monomers per protofibril. That means, if the labeled proteins bound substoichiometrically, the actual K_D_ values would be orders of magnitudes smaller than the apparent K_D_ because the actual seed concentration is lower than the monomer concentration. Together, the data suggest that 3R and 4R MAP2 effectively bind to tau fibrils.

### MAP2 captures tau fibrils from the solution

**Figure 4:**
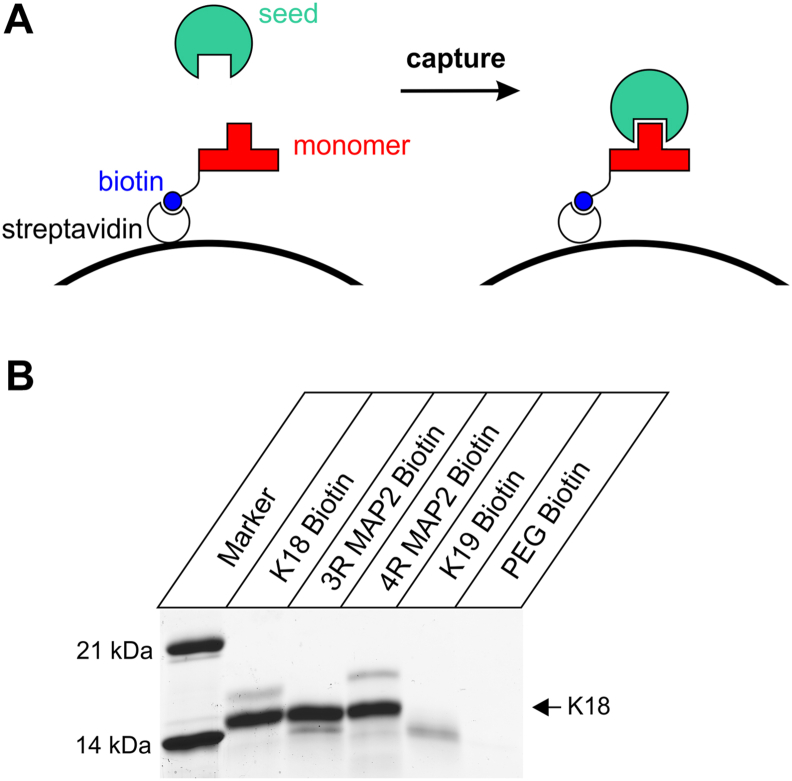
**MAP2 captures tau fibrils from the solution.***A*, schematic of fibril-pull-down assay. Biotinylated tau and MAP2 monomers are bound to immobilized streptavidin serving as bait for fibril capture. Boiling the complex in the SDS sample buffer leads to the dissociation of fibrils and the release of biotinylated monomers. *B*, biotinylated monomers of K18, 3R MAP2, 4R MAP2, and K19 were bound to streptavidin-conjugated magnetic beads, incubated with K18 fibrils, and then dissociated. Streptavidin beads linked to biotin PEG alone served as a negative control. All samples were analyzed by SDS-PAGE and Coomassie staining. Molecular weight markers are shown in the left lane. An *arrow* on the right points to the K18 monomers released upon fibril dissociation.

To obtain additional evidence for this interaction, a new pull-down assay was developed. The expectation was that biotinylated MAP2 monomers that are immobilized on magnetic streptavidin beads should be able to capture tau fibrils from the solution (Fig. 4*A*). An added advantage of this assay is that MAP2 will be monomeric when it is linked to the beads since the cofactor is absent during the cross-linking reaction.

In a first step, the natural cysteines of tau (K18 and K19) and MAP2 (3R and 4R) were replaced by serines, and single cysteines were introduced at the N termini. The proteins were then labeled with Maleimide-PEG_11_-Biotin. The polyethylene glycol (PEG) spacer arm in this reagent was chosen to minimize potential steric constraints between the bait and the target. The biotinylated proteins were attached to streptavidin-conjugated magnetic beads, washed, and then incubated with K18 fibril seeds. Unbound seeds were washed off with buffer. The beads were then mixed with SDS sample buffer and incubated for 10 min at 95 °C to unfold the conjugated streptavidin and to release biotinylated monomers and bound seeds. Analysis by SDS-PAGE and Coomassie staining revealed that biotinylated K18, 3R MAP2, and 4R MAP2 were able to capture K18 seeds since in these cases a pronounced K18 monomer band is visible in the gel (Fig. 4*B*). Notably, biotinylated K19 was unable to capture fibrils (Fig. 4*B*), consistent with the observation that K19 does not grow onto K18 seeds (42). Similarly, beads that were only attached to biotin PEG (lacking the protein bait) failed to capture K18 fibrils (Fig. 4*B*). The results indicate that the capture of K18 fibrils is based on specific interactions between the fibrils and the monomers. Notice that all biotinylated proteins are visible in the gel as faint bands with slightly higher molecular weights (K18 and 4R MAP2) or lower molecular weights (3R MAP2 and K19) than unmodified K18. This suggests that the proteins were indeed linked to streptavidin and only released upon boiling. In summary, the pull-down experiments corroborate that MAP2 monomers associate with tau fibrils.

### MAP2 binds to the end of tau fibrils

**Figure 5:**
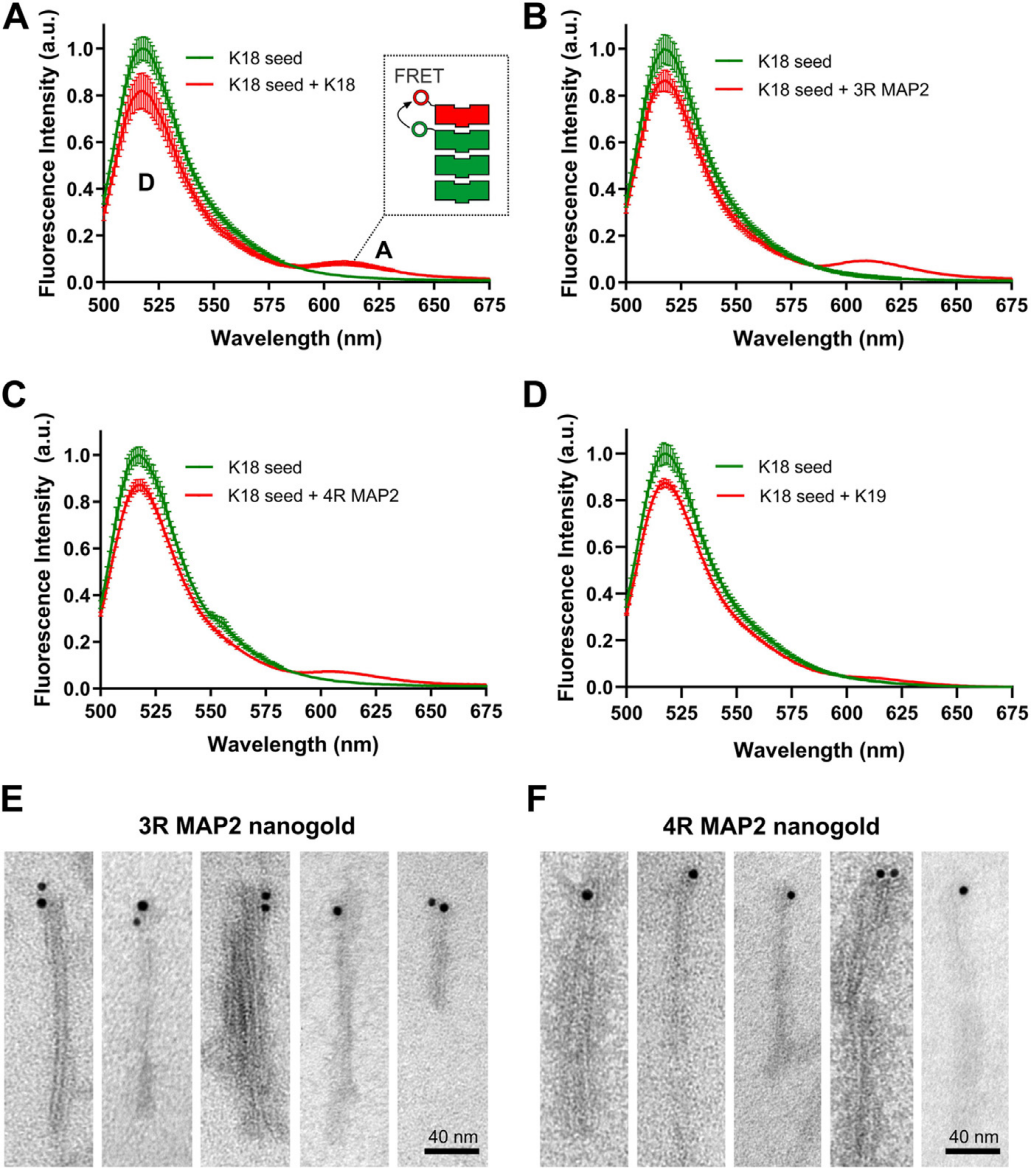
**MAP2 binds to the end of tau fibrils.***A*–*D*, K18 monomers (1 μM) labeled with the donor fluorophore Alexa488 were mixed with 10 μM K18 seeds and incubated for 1 h at 37 °C. To these seeds, monomers (1 μM K18, 3R MAP2, 4R MAP2, and K19) labeled with the acceptor fluorophore Alexa594 were added and incubated for an additional hour. The samples were excited at 488 nm and emission spectra recorded between 500 and 675 nm. Spectra were taken before (*green traces*) and after (*red traces*) the addition of acceptor-labeled K18 (*A*), 3R MAP2 (*B*), 4R MAP2 (*C*), and K19 (*D*). All measurements were performed in triplicate using independent seed batches. Error bars represent means ± SD. The emission peaks at 611 nm are indicative of FRET, suggesting that donor- and acceptor-labeled proteins bind to the end of the fibril, as highlighted schematically in *A* (inset). *E* and *F*, negative stain EM images of K18 seeds incubated with biotinylated MAP2 monomers and then with streptavidin-coated nanogold particles. Addition of 3R MAP2 is represented in (*E*), addition of 4R MAP2 in (*F*). Scale bars, 40 nm.

Although the previous experiments demonstrate an interaction between MAP2 monomers and K18 fibrils, the mode of interaction is not clear as monomers could either bind to the end of fibrils, along the fibrils, or both. To differentiate between these possibilities, we utilized fluorescence resonance energy transfer (FRET), a technique that is sensitive to the distances between appropriate donor and acceptor fluorophores (41). In a first step, K18 monomers were labeled with the donor fluorophore Alexa488 and incubated with a tenfold excess of seeds. This allowed for the incorporation of labeled K18 monomers at the end of the fibrils. In a second step, monomers of K18, 3R MAP2, and 4R MAP2 were labeled with the acceptor fluorophore Alexa594 followed by incubation with the donor-incorporated K18 seeds. The samples were excited at 450 nm and emission measured between 500 and 675 nm. In all cases, two emission peaks were observed (Fig. 5, *A*–*C*, red traces), one at 516 nm (donor peak) and another one at 611 nm (acceptor peak). The existence of the latter peak indicated that energy had transferred from the donor to the acceptor. Since the Förster radius for this pair is ∼6 nm (46) and energy transfer efficiency at longer distances drops precipitously, the data suggest that all three acceptor-labeled monomers (K18, 3R MAP2, and 4R MAP2) had added to the fibril end as schematically shown for K18 in the inset of Figure 5*A*. Notably, when acceptor-labeled K19 monomers were incubated with donor-incorporated K18 seeds, the FRET peak was largely absent (Fig. 5*D*), indicating little to no interaction of this protein with the fibril end. The FRET peak was also absent when donor-labeled tau seeds were measured alone (Fig. 5, *A*–*D*, green traces). Similarly, when mixtures of donor- and acceptor-labeled K18 and MAP2 were incubated in the absence of seeds, no FRET was observed (Fig. S6). The findings support the conclusion that a specific interaction between MAP2 and tau at the fibril end is the reason for FRET. A predominant interaction of MAP2 along the K18 fibril is unlikely since this should have abolished the FRET signal.

To independently verify these findings, we next incubated K18 fibril seeds with biotinylated MAP2 monomers and streptavidin-conjugated gold nanoparticles. The samples were visualized by negative stain electron microscopy. Gold nanoparticles were specifically localized to the fibril ends (Fig. 5, *E* and *F*). The combined data suggest that MAP2 caps tau fibrils.

### MAP2 inhibits tau aggregation in HEK293 cells

**Figure 6:**
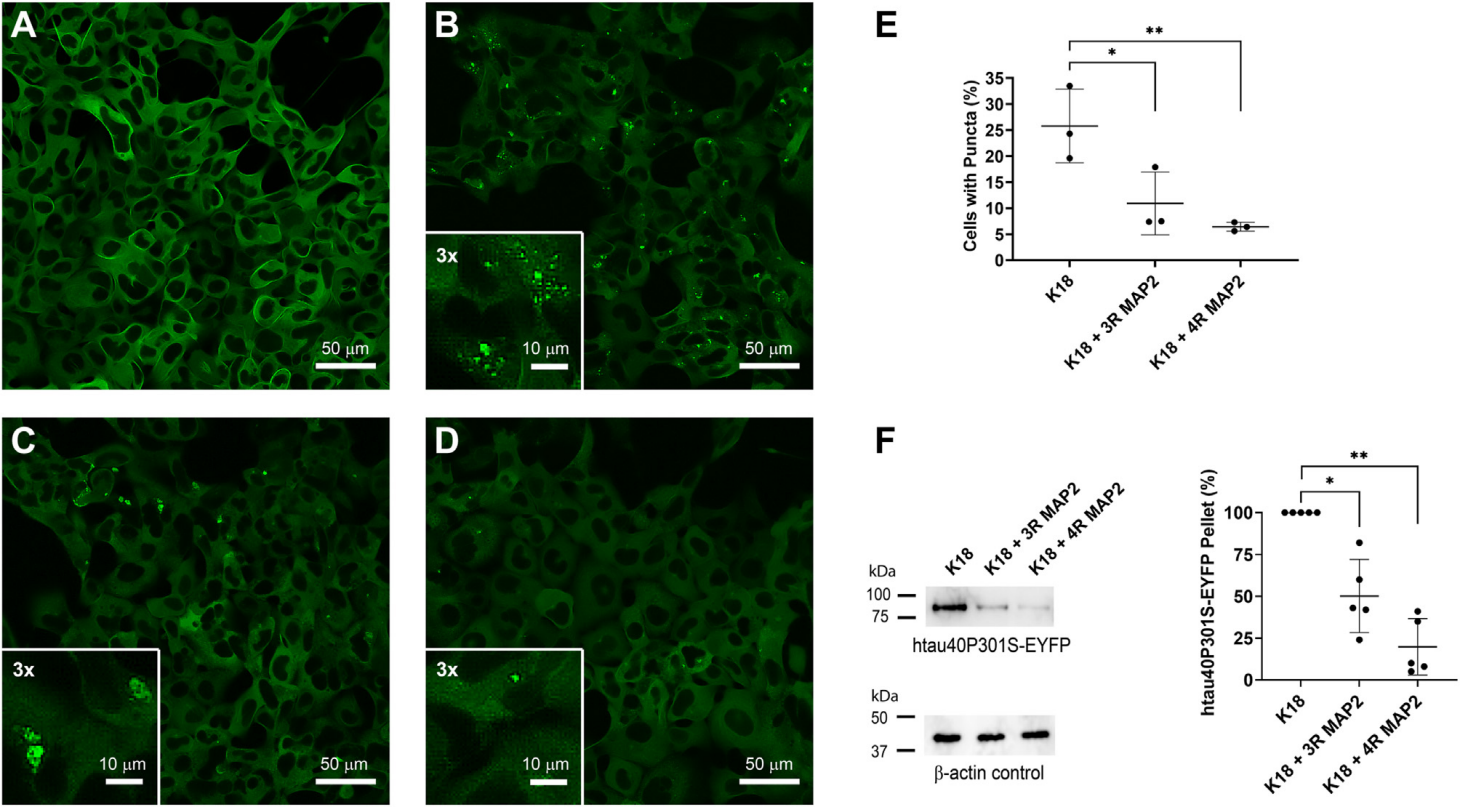
**MAP2 inhibits seeded tau aggregation in HEK293 cells.***A*–*D*, monoclonal HEK293 cells, stably expressing htau40P301S tagged with EYFP at the C-terminus, were transduced with buffer control (*A*), K18 seeds (*B*), K18 seeds preincubated with 3R MAP2 (*C*), or K18 seeds preincubated with 4R MAP2 (*D*) and imaged after 24 h of incubation. Scale bars, 50 μm. Insets in (*B*), (*C*), and (*D*) represent threefold magnified images with typical tau inclusions. Scale bars, 10 μm. *E*, quantification of puncta-containing cells. The bars represent the mean of biological triplicates with error bars representing SD. The number of cells analyzed for replicate experiments were as follows: K18 (N_1_ = 770, N_2_ = 559, and N_3_ = 985), K18 + 3R MAP2 (N_1_ = 778, N_2_ = 739, and N_3_ = 1220), and K18 + 4R MAP2 (N_1_ = 820, N_2_ = 721, and N_3_ = 1059). Statistical analysis was performed using ratio t tests. ∗*p* ≤ 0.1; ∗∗*p* ≤ 0.01. *F*, cells were extracted with 1% sarcosyl and aggregates sedimented for 70 min at 258,000*g*. Resuspended tau pellets were separated by SDS-PAGE and analyzed by Western blotting using the Tau-5 antibody (*upper left panel*). Total cell extracts were analyzed for β-actin to control for consistent protein concentration (*lower left panel*). Tau sedimentation experiments were carried out for biological quintuplicates. Band intensities were quantified densitometrically and analyzed by ratio *t* tests (*right panel*). ∗*p* ≤ 0.1; ∗∗*p* ≤ 0.01. Error bars represent means ± SD.

In a next set of experiments, we sought to determine whether MAP2 is capable of inhibiting tau seeding in a cellular context. There are several model systems that report on the formation of intracellular tau inclusions upon the addition of exogeneous seeds. We generated a monoclonal cell line of the human embryonic kidney 293 (HEK293) cells, expressing the P301S mutant of htau40, C-terminally tagged to enhanced yellow fluorescent protein (47) (see Experimental procedures and Fig. S7). This mutant was chosen because previous reports indicated that different from wildtype tau, it can be robustly seeded by exogenous fibrils (48). K18 seeds were preincubated with or without equimolar concentrations of MAP2 (3R and 4R) and then transduced into HEK293 cells expressing the P301S mutant. Transduction with buffer only served as a control. In the absence of exogenous seeds, no intracellular inclusions were observed (Fig. 6*A*) consistent with previous observations (49). In the presence of K18 seeds, about 25% of the cells contained inclusions, visible in the shape of puncta (Fig. 6*B*). The number of puncta-containing cells was markedly smaller when K18 seeds were preincubated with either 3R MAP2 (Fig. 6*C*) or 4R MAP2 (Fig. 6*D*). Also, there was some variability in the size and location of puncta (Fig. 6, *C* and *D*). Quantification revealed that the number of cells containing tau puncta was reduced from 25% in the absence of MAP2 to 11% in the presence of 3R MAP2 and 6.4% in the presence 4R MAP2 (Fig. 6*E*). To further verify the inhibitory role of MAP2, the HEK293 cells were treated with 1% sarkosyl, a detergent that solubilizes lipid membranes, but leaves tau fibrils intact (50). The fibrils were then sedimented, monomerized, and quantified using the Tau-5 antibody (Fig. 6*F*). In the presence of MAP2, a significantly smaller quantity of tau aggregates was observed in the pellets, mirroring the cell imaging data for tau puncta. Together, the findings suggest that in this model system, both variants of MAP2 inhibit the recruitment of tau monomers into fibrils.

### MAP2 inhibits the elongation of full-length tau fibrils

**Figure 7:**
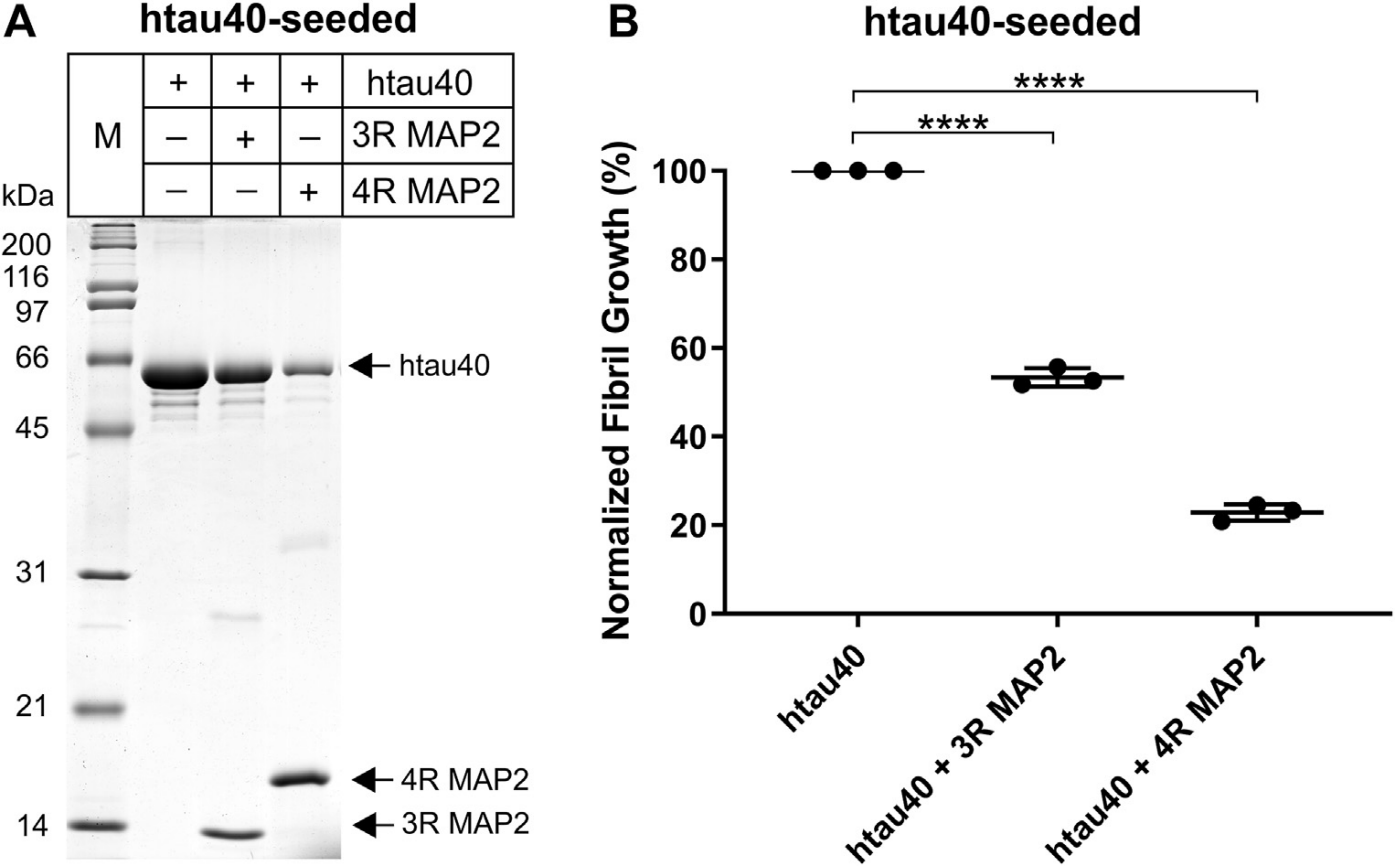
**MAP2 inhibits seeded aggregation of full-length tau fibrils.***A*, 10 μM htau40 monomers were mixed with 1% htau40 seeds (monomer equivalents) and incubated quiescently at 37 °C for 21 h. To test for inhibition of fibril growth, the samples were co-incubated with 10 μM 3R MAP2 or 10 μM 4R MAP2. The reactions were sedimented and analyzed by SDS-PAGE and Coomassie staining. The experiments were performed in triplicate (independent seed batches). A representative gel is shown. M = Protein marker. *B*, quantification of fibril growth using Image J. For each seed batch, the growth percentages are calculated with respect to the reaction that did not contain MAP2 set to 100%. Statistical comparison was performed using a paired *t* test. ∗∗∗∗*p* ≤ 0.0001. Error bars represent means ± S.D.

Previously, it was demonstrated that full-length tau fibrils are structurally distinct from K18 fibrils (51). Most notably, residues in the second half of repeat 4 are stacked in htau40 fibrils, yet largely disordered in K18 fibrils. To determine whether these and other potential conformational differences in the fibrils may have an effect on the inhibitory function of MAP2, we carried out in-vitro seeding experiments using full-length tau fibrils. First, htau40 seeds (1%) were incubated with htau40 monomers (10 μM) in the presence or absence of equimolar concentrations of MAP2 (3R and 4R). Then, the material was sedimented and analyzed by SDS-PAGE and Coomassie staining (Fig. 7*A*). Based on band intensities, tau fibril growth in the presence of 3R MAP2 and 4R MAP2 was reduced to 56% and 25%, respectively (Fig. 7*B*). The data reveal that MAP2 inhibits the elongation of full-length tau fibrils.

### MAP2 inhibits the amplification of AD tau filaments

**Figure 8:**
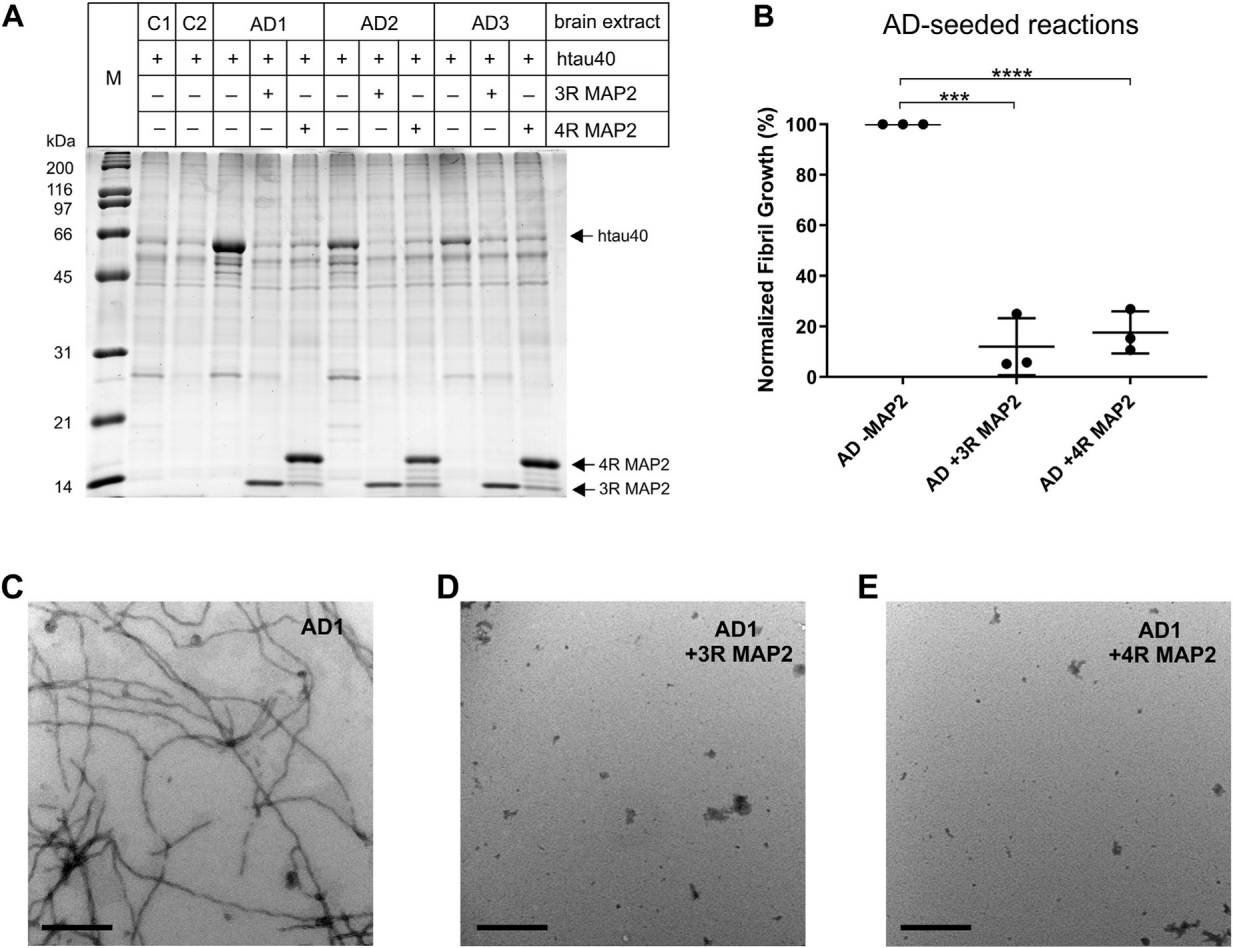
**MAP2 inhibits the amplification of tau fibrils from AD tissue.***A*, 30 μg brain homogenate was mixed with 5 μM htau40 monomer, 20 μM heparin, and 1 mM TCEP in an assembly buffer. Reactions probing for inhibition of tau aggregation also included 5 μM 3R MAP2 or 5 μM 4R MAP2. The samples were subjected to 30 h of consecutive 10 min cycles in a BGM FLUOstar Omega plate reader set to 37 °C. Each cycle consisted of 1 min double orbital shaking at 700 rpm followed by 9 min quiescent incubation. The samples were pelleted and analyzed by SDS-PAGE and Coomassie staining. M = Protein marker, C1, C2 = brain samples from non-demented controls, AD1-AD3 = brain samples from AD subjects (see Table S1). *B*, quantitative analysis of fibril amplification from AD extracts. The percentages of growth for reactions with MAP2 were calculated with respect to the reactions from the same AD sample that did not contain MAP2. Statistical comparison was performed using a paired *t* test. ∗∗∗*p* ≤ 0.001; ∗∗∗∗*p* ≤ 0.0001. Error bars represent means ± S.D. *C*, EM images of amplified tau fibrils from AD1 extracts in the absence of MAP2. *D* and *E*, EM images of amplified tau fibrils in the presence of 3R MAP2 and 4R MAP2, respectively. Scale bars, 500 nm.

Up to this point, the experiments utilized synthetically formed tau fibrils. These fibrils are ensembles of different conformers (51, 52, 53, 54, 55). Pathological tau filaments, in contrast, are mostly homogeneous (10), with structures distinct from those of synthetic fibrils (54, 55). To determine whether MAP2 interferes with the elongation of pathological tau filaments, we used AD brain tissue as a source of tau filament seeds. Brain homogenates from three AD cases and two non-demented controls (Table S1) were combined with htau40 monomers and then subjected to repetitive cycles of fracture and growth, a procedure that results in the amplification of minute quantities of filaments (56, 57, 58). To test for inhibition, the same reactions were carried out in the presence of MAP2. The samples were pelleted by centrifugation and then analyzed on a gel. In the absence of MAP2, all three AD samples resulted in tau amplification as revealed by the enhanced htau40 bands (Fig. 8*A*). Neither the control samples nor the AD samples containing MAP2 produced htau40 bands with comparable intensity. Notice that for 4R MAP2, a few minor degradation bands are observed in the gel (Fig. 8*A*). Densitometric analysis revealed that tau amplification in AD samples was reduced by over 80% when either one of the MAP2 variants (3R or 4R) was present (Fig. 8*B*). EM micrographs confirmed that the amplified aggregates in AD samples were of fibrillar nature (Figs. 8*C* and S8, *A* and *B*). In the presence of MAP2 (Fig. 8, *D* and *E*) and in control samples (Fig. S8, *C* and *D*), filaments were largely absent. Combined, the data suggest that tau amplification is specific for AD samples and that MAP2 inhibits this amplification.

## Discussion

**Figure 9:**
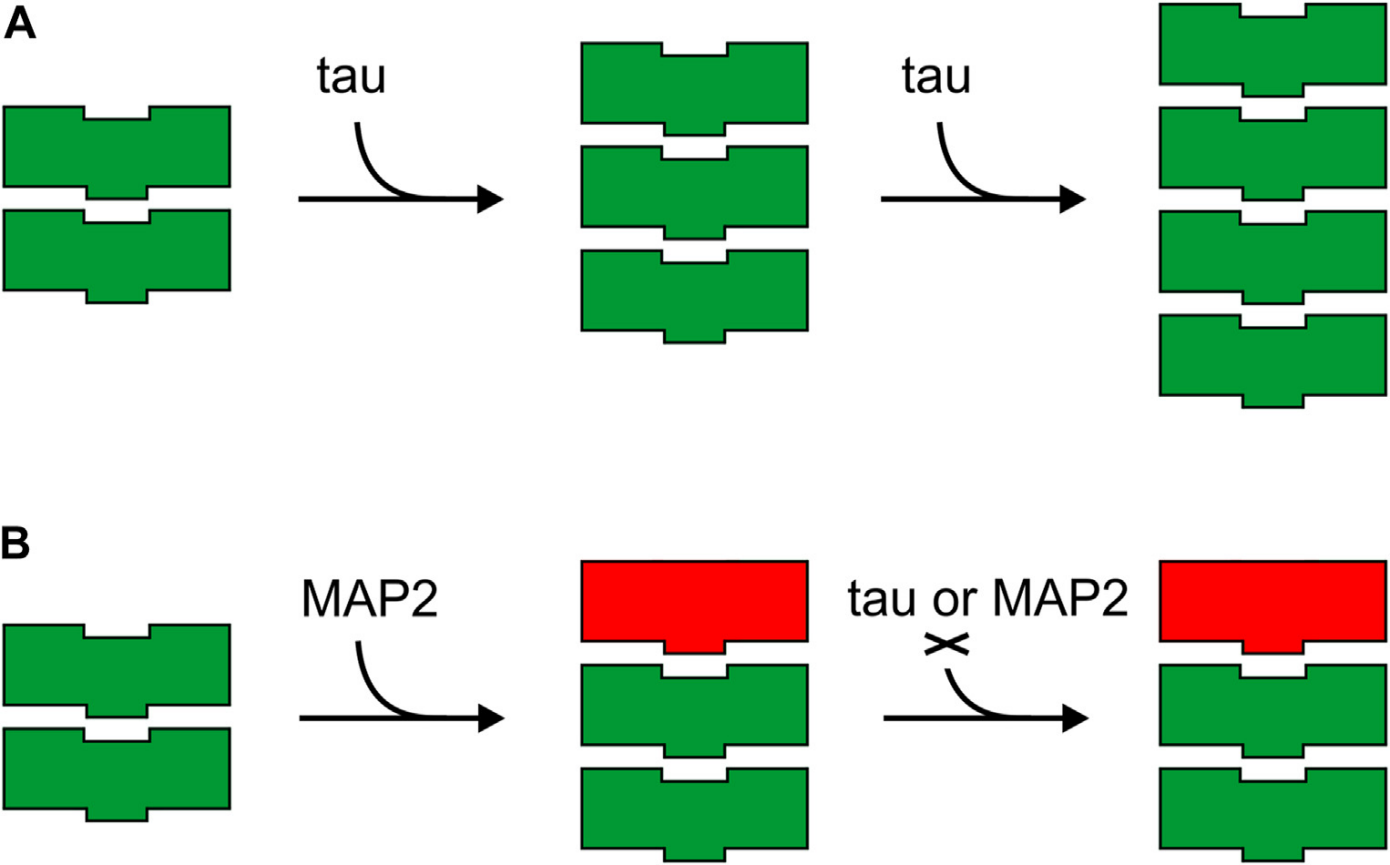
**Model of tau fibril growth and inhibition by MAP2.***A*, in the absence of MAP2, tau monomers (*green*) are recruited onto the fibril end. Each incoming monomer assumes the same conformation as the template, allowing the fibril to extend. *B*, when MAP2 monomer (*red*) binds to the fibril end the structure is altered, generating an acceptor surface that is incompatible with fibril growth. Now, neither tau nor MAP2 can be recruited onto the fibril.

Tau fibrillization is a key pathological hallmark of numerous fatal neurodegenerative disorders (59, 60). Understanding how this process is modulated by other proteins in the cell could offer new strategies for interfering with tau propagation in the human brain. Previously, it was observed that MAP2 associates with smaller tau aggregates (37). However, the nature of this interaction and the implications for tau aggregation have not been explored. In the present study, we set out to investigate the interactions between tau protein and MAP2 and to elucidate its potential impact on tau fibrillization. We discovered that 3R MAP2 and 4R MAP2 inhibit both spontaneous and seeded aggregation of tau. The inhibitory effect of MAP2 on tau seeding was observed in different experimental settings: *in vitro*, in HEK293 cells, and in AD brain extracts. MAP2 binds to the end of tau fibrils without further extending them, suggesting that MAP2 is unable to propagate the amyloidogenic state. These findings are consistent with previous work by Alonso *et al.* (37) which suggested that small AD P-tau seeds sequestered MAP2 without the concurrent formation of long filaments. The data are also compatible with the observation that MAP2 is not a major component of tau filaments in neurofibrillary tangles (61, 62). Long tau filaments that are comprising these tangles have a significantly smaller number of ends per mass than short soluble fibrils; therefore, recruitment of MAP2 would be highly reduced. Most importantly, the current study demonstrates that the binding of MAP2 to the fibril tip also abrogates the recruitment of tau monomers. MAP2 thus generates a surface incompatible with the binding of further tau or MAP2 monomers (Fig. 9). But what is the molecular basis for this inhibition?

Two types of interactions can be distinguished at the fibril tip: homotypic and heterotypic. When tau monomers dock onto the fibril end, the same residues stack on top of each other. These interactions are homotypic since the molecules are identical in sequence. The incoming monomers are perfectly suited for the core structure as each residue assumes its proper position, fitting onto the template like a piece into a puzzle. Given the proper supply of reactants, the fibrils could grow indefinitely, unless halted by a major misincorporation event. When MAP2 docks onto the fibril tip there will be sequence mismatches between the MAP2 monomer and the tau template. The interactions between these non-equal, yet homologous molecules are heterotypic. Although much more work is needed, it appears that MAP2 has favorable interaction energies with the fibril tip, but unfavorable elongation energies once bound. In molecular terms, this means that the incoming molecule can form a stable outer layer. Additional layers, however, are not possible since mismatching side chains may interfere with stacking and cross-sectional β-sheet packing. Notably, short peptides with altered amino acid sequences that target the cross-sectional interfaces of aggregation-prone regions have been successfully employed in the capping of various amyloid fibrils and the inhibition of seeding (63, 64, 65, 66). Recently, Louros *et al.* (67) conducted an extensive study on the heterotypic amyloid interactions of small peptides and the contributions of individual amino acids. Amongst others, it was found that aromatics and long positively charged side chains were effective in facilitating heterotypic capping due to steric clashes and charge repulsions that countered further elongation. The destabilization of cross-sectional interfaces was identified as a major contributing factor (67). Related to these findings, inward-pointing charged side chains in edge strands are known to be a common negative design principle that prevents aggregation of soluble β-sandwich proteins (68) and has been employed to convert *de novo* amyloid fibrils into monomeric β-sheet proteins (69). Other negative design principles include the use of bulges, prolines, and loops at the edges of β-sheets. Although it is currently unknown which residues in MAP2 are responsible for the capping of tau fibrils, it is likely that multiple mechanisms are at play. It is also important to recognize that the interactions between MAP2 and Tau fibrils could be modulated by posttranslational modifications. Phosphorylation of specific side chains, for example, could alter the affinity between the proteins at the fibril/monomer interface or affect the conformational ensemble of MAP2 (70, 71) and thereby the exposure of the repeat region. The latter mechanism might be particularly relevant for full-length MAP2, where the overall shape is determined by transient interactions between the acidic N-terminus and the positively charged C-terminal region (71).

Tau fibrils assume a plethora of different folds with varying β-sheet interactions and core sizes (11). Hence, the exposed contact surfaces at the fibril tips can vary widely. The fibrils that were employed in the current study are known to have different folds. While filaments from AD tissue are characterized by a single conformer (72), fibrils formed in the presence of heparin are heterogeneous ensembles (51, 52) with structures varying from those in AD (54, 55). MAP2 inhibited the seeding of all tau fibrils studied suggesting that it engages with a broad spectrum of conformers. However, it is likely that there are context-specific differences in the interactions that could modulate the affinities of MAP2. Another important aspect of capping is that the two ends of the fibril are asymmetric. Our binding data (Fig. 3) were best fit with a one-site binding model, suggesting that MAP2 may preferentially interact with one end of the fibril. This would be in line with the previously described asymmetric growth of amyloid fibrils (73). However, the growth characteristics of tau at the two fibril ends are still poorly understood. It is very well possible that there could be conformation-dependent differences in growth, as reported in yeast prions (74), that extend to the MAP2-tau fibril interaction.

An important question remaining is what is the biological implication of the herein-described findings? Whereas the sequestering of MAP2 compromises microtubule stability (37), the inhibition of spontaneous and seeded tau aggregation could have a more direct impact on pathogenic tau species. Additional research is needed to determine how MAP2 may contribute to the selective cellular vulnerability observed in Alzheimer’s disease and other tauopathies (75, 76). The presented work should raise interest in MAP2 as a modulator of tau pathology and a potential new therapeutic target.

## Experimental procedures

### Tau and MAP2 constructs

The largest human tau isoform htau40 and the truncated three- and four-repeat versions K18 and K19 were previously cloned into pET-28 (44). All site-directed mutagenesis was performed using the QuikChange method following the manufacturer’s (Agilent) instructions. Constructs containing the microtubule-binding repeats of MAP2 (residues 362–490 according to UniProt: P11137-4) were synthesized by Biomatik and cloned into pET-28 using the NcoI/XhoI cloning sites. This created two additional residues (MetGly) at the N-terminus. Constructs of K18, K19, 3R MAP2, and 4R MAP2 with a single cysteine at position 3 (MetGlyCys….) were generated in the same manner. Latter constructs had the natural cysteines replaced by serines (one cysteine in three-repeat constructs and two cysteines in four-repeat constructs). The mammalian expression construct htau40P301S-EYFP (Fig. S7) was synthesized by GenScript and cloned into pcDNA3.1 using the HindIII/XhoI restriction sites.

### Protein expression and purification

For bacterial protein expression, pET-28 vectors containing the respective MAP2 and tau inserts were transformed into *Escherichia coli* strain BL21 (DE3) and then cultured on LB (Miller) agar plates. Single colonies were transferred into LB medium and incubated under agitation for 16 to 18 h at 37 °C. The cultures were diluted 1:100 with LB medium and again incubated while agitating at 37 °C until the optical density at 600 nm reached a value of 0.7 to 1.0. For the selection of transformed bacteria, the medium contained kanamycin (50 μg/ml in agar plates and 20 μg/ml in solution). Protein expression was induced with 0.5 to 1 mM IPTG for 3.5 h at 37 °C, shaking. The cells were harvested at 5500*g* for 10 min. Pellets were taken up in 500 mM NaCl, 20 mM piperazine-N, N′-bis (2-ethanesulfonic acid) (PIPES, JT Baker or RPI) pH 6.5, 5 mM ethylenediaminetetraacetic acid (EDTA, Fisher), 50 mM 2-mercaptoethanol (Fisher Scientific), and stored at −80 °C until further use. Protein purification was initiated by heating the resuspended cells for 20 min at 80 °C followed by tip-sonication for 1 min on ice at 50% power. The samples were then centrifuged for 30 min at 20,000*g* to separate soluble tau or MAP2 from insoluble cellular debris. The supernatant was adjusted to 55% (w/v) ammonium sulfate and rocked for 12 to 16 h at 22 °C to precipitate the remaining soluble protein. Precipitated protein was collected by centrifugation at 20,000*g* for 10 min and taken up in either H_2_0 (tau proteins) or 10 mM PIPES pH 6.5, 50 mM NaCl, 2 M urea (MAP2 proteins) both supplemented with 4 mM DTT. Samples were sonicated for 1 min at 50% power, syringe filtered (Pall Acrodisc 0.45 μM), and diluted with H_2_O until the conductivity was below 20 mS/cm. Protein was loaded onto a cation exchange column (Mono S 10/100 Gl, GE Healthcare) using 50 mM NaCl, 20 mM PIPES pH 6.5, 0.5 mM EDTA and eluted with a linear gradient of 1 M NaCl, 20 mM PIPES, and 0.5 mM EDTA. Proteins were pooled based on SDS-PAGE analysis and further purified by size exclusion chromatography. A Superdex 75 column (GE Healthcare) was used for truncated tau and MAP2, a Superdex 200 column (GE Healthcare) was used for full-length tau. The proteins were eluted from either column with buffer containing 100 mM NaCl, 20 mM Tris, pH 7.4, 1 mM EDTA, and 2 mM DTT. Protein fractions were pooled and precipitated overnight at 4 °C using an equimolar volume of methanol (precipitation of htau40) or three-fold volumetric access of acetone (precipitation of all other proteins) along with 4 mM DTT. Precipitated protein was collected by centrifugation for 10 min at 12,000*g*, washed with methanol or acetone containing 2 mM DTT, aliquoted, and stored at −80 °C.

### Protein solubilization and labeling

Protein pellets were dissolved in 8 M guanidinium HCl (Thermo Scientific). For cross-linking reactions, a tenfold molar excess of maleimide-modified label (Alexa 488 Fluor (Thermo Scientific, Cat# A10254), Alexa 594 Fluor (Thermo Scientific, Cat# A10256), ATTO633 (ATTO-TEC GmbH, Cat# AD 633-41), or PEG-biotin (Thermo Scientific, Cat# 21911)) was added. The samples were incubated for 2 to 24 h at 22 °C. To remove excess label and/or denaturant, the samples were passed over PD-10 columns (GE Healthcare) equilibrated with assembly buffer (10 mM HEPES pH 7.4, 100 mM NaCl, 0.1 mM NaN_3_). Protein concentrations were determined using the bicinchoninic acid assay (BCA, Pierce).

### Fibril formation and seed generation

Fibrils to be used as seeds were formed by incubating a 500 μl-mixture of 25 μM tau, 50 μM heparin (Celsus, average molecular weight 4400 Da, Cat# EN-02912), 0.5 to 1 mM tris(2-carboxyethyl)phosphine (TCEP; Gold Biotechnology) and assembly buffer while stirring with a Teflon-coated micro stir bar (5 × 2 mm) for 3 days at 220 rpm and 22 °C (K18) or for 3 to 4 days at 160 rpm and 37 °C (htau40). To generate seeds, fibrils were subjected to 30 s sonication on ice at 20% power with a Fisher Scientific sonifier (model 100 with a 2 mm tip).

### Inhibition of fibril elongation by MAP2 followed by sedimentation

Seeds (1% monomer molar equivalents of htau40 and 10% monomer equivalents of K18) were mixed with 10 μM K18 or htau40 monomer, 20 μM heparin, 0.5 to 1 mM TCEP and assembly buffer, and allowed to incubate quiescently at 37 °C for either 6 h (K18) or 21 h (htau40). Experiments probing the blockage of htau40 elongation included 10 μM 3R or 4R MAP2. Experiments probing the inhibition of K18 elongation included varying concentrations of 3R MAP2 (12.5 nM-25 μM) and 4R MAP2 (12.5 nM-15 μM) as specified in the result section. After incubation, the samples were centrifuged for 30 min at 130,000*g*. The pellets were separated from supernatants, volumes adjusted with sample buffer (62.5 mM TRIS pH 6.5 (Sigma), 4% SDS (J.T. Baker), 10% sucrose (MP Biomedicals), 5% 2-Mercaptoethanol, 1.5 mM Bromophenol Blue (Sigma)), and then boiled for 5 min. Equal amounts of samples were analyzed by SDS-PAGE (14–16%) and Coomassie staining. Band intensities were quantified by Image J. IC_50_ values were computed by applying the four-parameter nonlinear regression model in GraphPad Prism 9.

### Tau aggregation kinetics in the absence and presence of MAP2

To determine the kinetics of fibril elongation 10 μM K18 was mixed with 20 μM heparin, 0.5 mM TCEP, 5 μM ThT, and 3% seeds (monomer molar equivalent) in assembly buffer. ThT fluorescence was measured on a Tecan M1000 or Omega Fluorstar fluorescent plate reader. The samples were excited at 440 nm and emission measured at 480 nm over a period of 4 h while quiescently incubating at 37 °C. To assess the effects of MAP2 on fibril elongation, 2.5 μM 3R or 4R MAP2 were included in the original reaction mix.

The kinetics of tau aggregation in the absence of seeds was also measured by ThT fluorescence. Here, 25 μM K18 was mixed with 50 μM heparin, 0.5 mM TCEP, and 20 μM ThT in assembly buffer. Experiments that tested for inhibition included 3.2 μM 3R or 4 R MAP2 in the mixtures. Aggregation was monitored for 8 h at 37 °C. During this time the reactions were subjected to cycles of 9 min quiescent incubation followed by 1 min double orbital shaking at 700 rpm.

### Fluorescence anisotropy measurements

In a first step, 50 μM K18 monomers and 6.2 μM heparin were added to 5% K18 seeds, incubated for 16 h at 37 °C, and sonicated for 2 min at 20% power with a tip sonifier. The newly generated seeds (0–20 μM monomer equivalents) were then titrated into a solution containing 100 nM ATTO633-labeled protein (K18, K19, 3R MAP2 or 4R MAP2 labeled at the native cysteine in repeat 3) and equilibrated for 16 h at 22 °C. Fluorescence anisotropy was measured in a Fluorolog-3 spectrofluorometer (Horiba Scientific) equipped with automated polarizers. Excitation and emission wavelengths were 625 nm and 650 nm, respectively. Slit widths were set at 3 nm for excitation and 6 nm for emission. The G-factor was determined by the quotient,

(1) $$G=\frac{I_{HV}}{I_{HH}}$$

where *I*_*HV*_ and *I*_*HH*_ are the fluorescence intensities of the vertically and horizontally polarized emissions when the samples are excited with horizontally polarized light.

Upon excitation with vertically polarized light and measurement of the vertically and horizontally polarized emissions (*I*_*VV*_ and *I*_*VH*_, respectively), the anisotropy (r) was computed using the following equation.

(2) $$r=\frac{I_{VV}-GI_{VH}}{I_{VV}+2GI_{VH}}$$

Three measurements using independent batches of seeds were carried out for each binding reaction. The data were plotted in GraphPad Prism 9 and fit with a one-site binding model according to the following equation,

(3) $$r=r_{0}+\frac{{\Delta r}_{MS}{\left[S\right]}_{T}}{K_{d}+{\left[S\right]}_{T}}$$

where r_0_ is the initial anisotropy of the labeled monomer (M), [S]_*T*_ is the total concentration of seeds (monomer equivalents), *K*_*d*_ is the dissociation constant, and Δ*r*_*MS*_ is defined as (*r*_*MS*_ – *r*_*M*_) ([M]_*T*_), where *r*_*MS*_ and *r*_*M*_ are the anisotropies of the labeled monomer/seed complex and the labeled monomer, respectively.

### FRET measurements

To generate new tau fibrils, 50 μM K18 monomers were combined with 6.2 μM heparin, added to 5% K18 seeds, and incubated quiescently at 37 °C for 16 h. A volume of 500 μl of this sample was sonicated for 2 min at 20% power to produce small fibril seeds. Next, 10 μM seeds were added to 1 μM K18 monomers labeled on the N terminus with Alexa 488 and incubated quiescently at 37 °C for 1 h to allow for the recruitment of labeled protein onto the fibril ends. Excitation and emission slit widths were set to 5 nm. The samples were excited at 450 nm and emission spectra were collected from 500 to 675 nm. This was followed by the addition of 1 μM monomer (K18, K19, 3R MAP2, or 4R MAP2) labeled on the N terminus with Alexa 594 as an acceptor dye. These reactions were incubated for another hour at 37 °C. Following incubation with the acceptor species, emission spectra were taken using the same settings as above.

### Fibril pull-down experiments

Protein bait was installed by combining 250 μl of 20 μM PEG-Biotinylated protein (tau or MAP2) with 200 μg hydrophilic streptavidin-conjugated magnetic beads (New England BioLabs, Cat# S1421S) and incubating the mixture for 60 min at 22 °C while rotating at 40 rpm. The beads were then separated with a magnet, thoroughly rinsed with 1000 μl assembly buffer, and separated again. After resuspension in 250 μl assembly buffer containing 5 μM preformed K18 seeds (monomer equivalents), the beads were incubated for another 60 min while rotating at 40 rpm. Beads were collected, washed, and taken up in 50 μl of 1× SDS sample buffer. The samples were heated for 10 min at 95 °C, separated by SDS-PAGE, and stained with Coomassie R250.

### Fibril amplification from brain homogenate and blockage by MAP2

Frozen brain tissue from frontal cortex (AD and control, see Table S1) was provided by the Carrol A. Campbell, Jr Neuropathology Lab at the Medical University of South Carolina. The tissue was combined in a 1:10 (w/v) ratio with buffer containing 10 mM HEPES, 5 mM EDTA, 150 mM NaCl, 0.1% Triton X-100 (VWR International) and 1× Halt Protease Inhibitor (Thermo Scientific). This mixture was homogenized on ice for 5 min in a 25 ml Potter-Elv tissue grinder (Wheaton) using a SteadyStir Digital (Fisher Scientific) at 250 rpm, followed by 20 min centrifugation at 17,000*g* at 5 °C. Pellets were resuspended in the same initial buffer volume, then subjected to another 5 min 250 rpm homogenization. Total homogenate protein concentration was determined by the BCA assay. Aliquots were flash-frozen and stored at −80 °C.

Tau fibrils in AD brain homogenates were amplified using repetitive cycles of fracture and growth. Each reaction mixture included 5 μM htau40 monomer, 20 μM heparin, 1 mM TCEP, 30 μg tissue homogenate (AD or control), and assembly buffer. Frozen brain homogenate aliquots were diluted 1:10 with assembly buffer. Reactions probing the ability of MAP2 to block amplification of fibrils from AD tissue included 5 μM 3R MAP2 or 4R MAP2. Reactions were contained in a 96-well plate (Thermo Scientific) and subjected to 30 h of consecutive 10 min cycles in a BGM Labtech FLUOstar Omega plate reader set to 37 °C. Each cycle consisted of a 1 min double orbital shake at 700 rpm followed by a 9 min quiescent incubation. Following assay completion, samples were centrifuged for 30 min at 130,000*g*. The pellets were taken up in the same volumes of SDS sample buffer, heated for 5 min at 95 °C, and analyzed by SDS-PAGE (15%) and Coomassie staining. Band intensities were quantified by Image J.

### Transmission electron microscopy

Samples of amplified tau aggregates and amorphous MAP2 were diluted 1:1 with assembly buffer to a final concentration of 2.5 μM. Formvar/carbon-coated 200 mesh copper grids (Electron Microscopy Sciences) were placed for 1.5 min onto 10-μl droplets of these samples. Excess liquid was blotted on Whatman filter paper and the grids were placed for 1.5 min onto 10 μl-droplets of 2% uranyl acetate (Electron Microscopy Sciences, 0.2 μm syringe filtered). Grids were again blotted with filter paper and air-dried for another 5 min. Images were recorded with an FEI Tecnai T12 Biotwin transmission electron microscope at 100 KV using a Gatan CCD camera.

### Gold nanoparticle labeling

K18 fibrils (25 μM) were sonicated for 60 s at 20% power, combined with biotinylated 3R and 4R MAP2 (5 μM), and allowed to incubate for 24 h at 37 °C. The resultant solution was mixed 1:1 with streptavidin-coated gold nanoparticles (EM-grade 6 nm, Electron Microscopy Sciences cat# 25264) at a final dilution of 1:40 and incubated for 90 min. The samples were then prepared for negative stain TEM analysis as described above.

### Cell culture

HEK293 cells, purchased from American Type Culture Collection (ATTC number: CRL-1573) were grown in Dulbecco’s Modified Eagle Medium + GlutaMAX-1 (DMEM, Gibco) supplemented with 10% Fetal Bovine Serum (FBS, Gibco) and 40 U/ml of Pen Strep (Gibco). Cells were grown to 70% confluency with media changes every other day. All cells grew at 37 °C, 5% CO_2,_ in a humidified incubator. To passage cells, the media was aspirated, and the cells were washed with phosphate-buffered saline (PBS pH 7.4, Gibco). Incubation for 5 min with TrypLE Express Trypsin (Gibco) resulted in the release of cells from the plates. Protease activity was suppressed by the addition of 10% FBS in DMEM. The suspension was then analyzed for cell density using a Bright Line Hemocytometer (Hausser Scientific) and plated at a density of 0.3 × 10^6^ cells/well into 8-chamber cell culture slides (CELLTREAT) for transfection.

### Transfection of HEK293 cells and selection of monoclonal lines

Plasmid (pcDNA3.1) containing htau40P301S-EYFP was transfected into HEK293 cells using Lipofectamine 2000 Transfection Reagent (Thermo Scientific) following the manufacturer’s instructions. After 24 h, the media was replaced with 10% FBS in DMEM containing 700 μg/ml of G418 (Geneticin) antibiotic to allow for the selection of cells containing the plasmid. A cell death curve indicated that at this concentration of antibiotic, non-transfected cells were not viable. After 1 week under selection media, cells were plated in a 96-well plate for confocal imaging to confirm stable transfection of htau40P301S-EYFP. Cells were re-plated onto 100 × 20 mm culture plates (Nunc Easydish, Thermo Scientific) and grown to 70% confluency. Non-transfected HEK293 cells were also plated as a control. Cells were then harvested using TrypLE Express Trypsin (Thermo Scientific). Once cells were released from the plate, PBS containing 1% FBS was used to halt TrypLE Express Trypsin activity. The cells were transferred into a 15 ml conical vial, pelleted by centrifugation at 300*g*, and washed twice with 1% FBS in PBS before being resuspended in 1 ml of 1% FBS in PBS and filtered through a Sterile Cell Strainer (40 μm Nylon Mesh, Fisher). The cells were passed through a Sony FX500 Exchangeable Fluids Cell Sorter to select for single cells expressing htau40P301S-EYFP. Single cells were sorted into a 96-well plate containing 200 μl of 10% FBS in DMEM supplemented with 40 U/ml of Pen Strep per well and incubated for 1 week with regular media changes until individual colonies of cells could be confirmed using an inverted microscope. Cells were grown to 70% confluency before being split into 2 ml culture flasks and then into 96-well plates. Monoclonality was confirmed by confocal microscopy using an Olympus IX83 microscope. In total, eight monoclonal cell lines were generated; one of them (Mono4) was used for further experiments.

### Inhibition of tau aggregation in cell culture assayed by puncta formation

Monoclonal htau40P301S-EYFP HEK293 cells (Mono4) were plated at 10,000 to 20,000 cells per well in a 96-well plate containing 10% FBS in DMEM supplemented with 700 μg/ml of G418 and grown to 70 to 100% confluency at a total volume of 210 μl. To test for inhibition of seeding, K18 seeds (20 μM) were incubated for 1 h at 37 °C with either 3R MAP2 (11 μM), 4R MAP2 (11 μM), or buffer (100 mM NaCl, 10 mM HEPES, pH 7.4) as a control, mixed with Opti-MEM and Lipofectamine 2000 and then added directly to cell media to achieve a seed concentration of 1.1 μM. Cells were incubated with seeds for 24 h before imaging on an Olympus Fluoview FV3000 Confocal Microscope with a 488 nm laser. The experiments were repeated with three independent seed batches. Images of cells with and without puncta were quantified *via* ImageJ software (National Institutes of Health) and plotted with GraphPad using a ratio paired *t* test.

### Inhibition of tau aggregation in cell culture assayed by Western blotting

Cells were plated in 6-well plates (CELLTREAT) at 100,000 to 200,000 cells per well and grown to 70 to 100% confluency. The addition of K18 seeds (preincubated with buffer or MAP2) was performed as described above. After 24-h incubation, cells were washed with 1 ml PBS (2–3 min) and then incubated for 5 min at 22 °C with 300 μl lysis buffer (1% sarkosyl, 10 mM TRIS-HCl, 150 mM NaCl, Halt Protease Inhibitor Cocktail (Thermo Scientific; Cat#78430), 1 mM EGTA, and 5 mM EDTA at pH 7.4). Lysates were pooled (3 wells per condition) and syringe-sheared five times with a 27-gauge needle. A BCA assay was employed to determine protein concentrations and adjust accordingly. Samples were centrifuged for 70 min at 258,000*g* to separate tau aggregates from supernatants. Tau pellets were resuspended in 1× sample buffer, heated for 5 min at 96 °C, and loaded onto a 12% SDS PAGE. Proteins were transferred to PVDF membranes using a Semi-Dry Transfer Cell (Bio-Rad). Membranes were blocked for 1 h with 5% BSA (Sigma)/TBS (20 mM TRIS, 137 mM NaCl, and 0.1% Tween 80 at pH 7.6), incubated for 1 h with primary Tau-5 antibody (Thermo Scientific; Cat# PIMA512808; 1:400 dilution), washed, incubated for 1 h with secondary mouse IgGκ light chain binding protein conjugated to HRP (Santa Cruz, Cat# sc-516102; 1:1000 dilution), and washed again. The membranes were then incubated for 1 min with SuperSignal West Femto Maximum Sensitivity Substrate (Thermo Scientific) and imaged. Protein bands were quantified using ImageJ and data were plotted in GraphPad using a ratio paired *t* test. To independently verify that the cell lysates that were used for assessing tau seeding contained the same overall protein concentrations, the adjusted cell lysates (above) were separated by 12% SDS-PAGE, blotted onto PVDF membranes, and analyzed using beta-actin rabbit polyAB (Proteintech, Cat# 20536-1-AP; 1:1000 dilution) as a primary antibody and goat anti-rabbit IgG(H + L), HRP conjugate (Proteintech, Cat# SA00001–2; 1:2000) as a secondary antibody.

## Data availability

All data are contained within the article.

## Supporting information

This article contains supporting information.

## Conflict of interest

The authors declare that they have no known competing financial interests or personal relationships that could have appeared to influence the work reported in this paper.
