# Supplementary material for: MAP2 caps tau fibrils and inhibits aggregation
Source: J Biol Chem. 2023 Jun 5;299(7):104891. doi: 10.1016/j.jbc.2023.104891 (PMC10404690; doi:10.1016/j.jbc.2023.104891)
Supplement: Supporting Figures S1–S8 and Table S1 [file mmc1.pdf]

# SUPPORTING INFORMATION

## MAP2 caps tau fibrils and inhibits aggregation

**Michael R. Holden[‡], Brad J. Krzesinski[‡], Hilary A. Weismiller[‡], Justin R. Shady, and Martin Margittai***

From the Department of Chemistry and Biochemistry, University of Denver, Denver, CO 80208, USA

Running title: MAP2 caps tau fibrils

[‡]These authors contributed equally
*To whom correspondence should be addressed: Martin Margittai, Department of Chemistry and Biochemistry, University of Denver, 2190 East Iliff Ave, Denver CO 80208. Tel: (303)-871-4135; Fax: (303)-871-2254. E-mail: martin.margittai@du.edu

## Contents

**Figure S1.** 3R MAP2 inhibits K18 fibril elongation.

**Figure S2.** 4R MAP2 inhibits K18 fibril elongation.

**Figure S3.** MAP2 forms amorphous aggregates.

**Figure S4.** Heparin titration does not rescue tau aggregation.

**Figure S5.** MAP2 monomers, not aggregates, inhibit tau fibril growth.

**Figure S6.** In the absence of seeds, tau and MAP2 produce no FRET signal.

**Figure S7.** Mammalian expression construct of tau used in this study.

**Figure S8.** Tau fibrils can be amplified from AD brain extracts, but not from control extracts.

**Table S1.** Brain tissue used in this study.

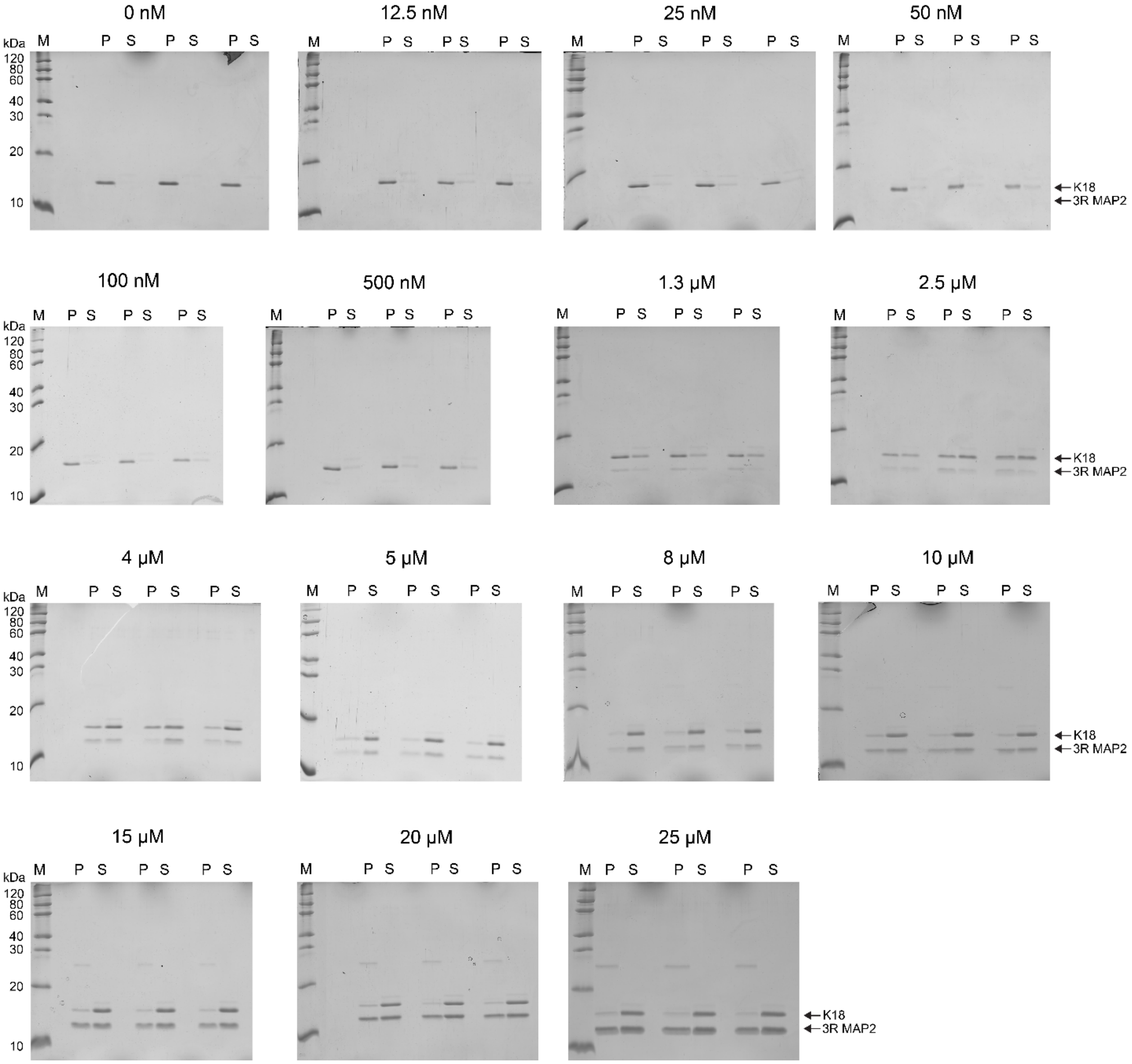


**Figure S1. 3R MAP2 inhibits K18 fibril elongation.** 10 µM K18 monomers were mixed with 10% seeds (monomer equivalents) and incubated for 6 h at 37 °C in the presence of increasing concentrations of 3R MAP2. The samples were then sedimented for 30 min at 130,000 x g and analyzed by SDS-PAGE and Coomassie staining. Each experiment was carried out in triplicate. The data were used to determine the $IC_{50}$ value for 3R MAP2-mediated inhibition of tau aggregation (Fig. 2D).

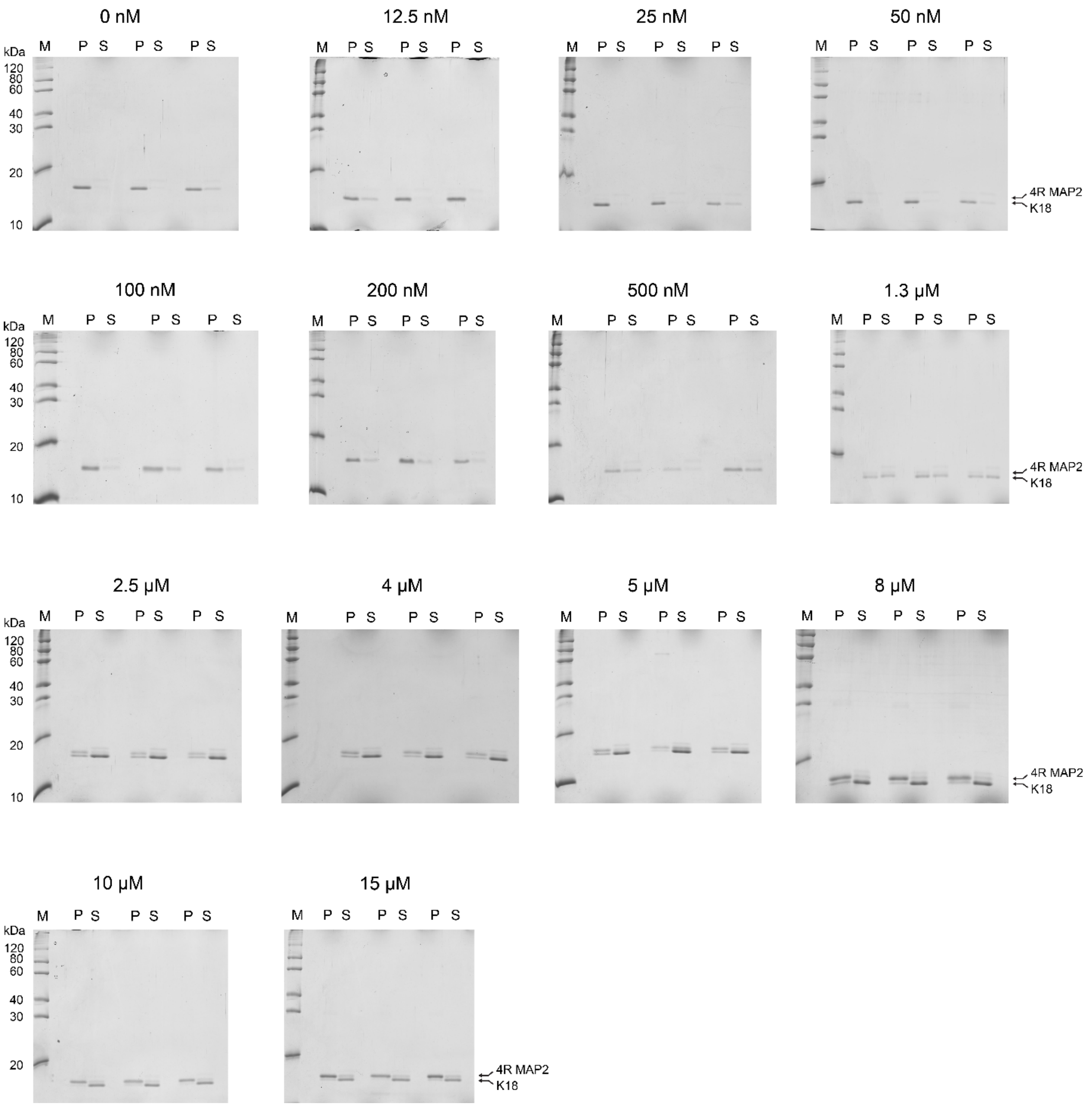


**Figure S2. 4R MAP2 inhibits K18 fibril elongation.** 10 µM K18 monomers were mixed with 10% seeds (monomer equivalents) and incubated for 6 h at 37 °C in the presence of increasing concentrations of 4R MAP2. The samples were then sedimented for 30 min at 130,000 x g and analyzed by SDS-PAGE and Coomassie staining. Each experiment was carried out in triplicate. The data were used to determine the $IC_{50}$ value for 4R MAP2-mediated inhibition of tau aggregation (Fig. 2E).

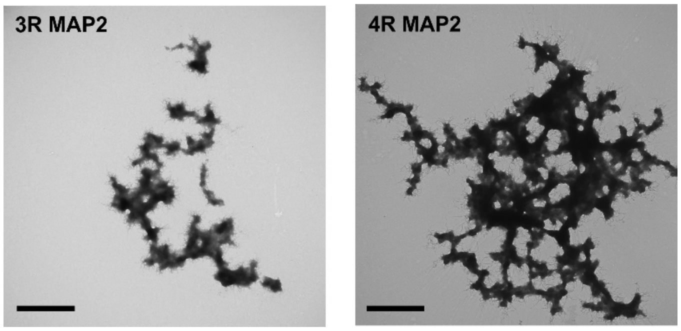


**Figure S3. MAP2 forms amorphous aggregates.** Monomers of 3R MAP2 or 4R MAP2 (5 μM) were incubated for 16 h at 37°C in the presence of heparin (10 μM). Shown are representative negative stain images of amorphous aggregates of 3R MAP2 (left panel) and 4R MAP2 (right panel). Scale bars, 2 μm.

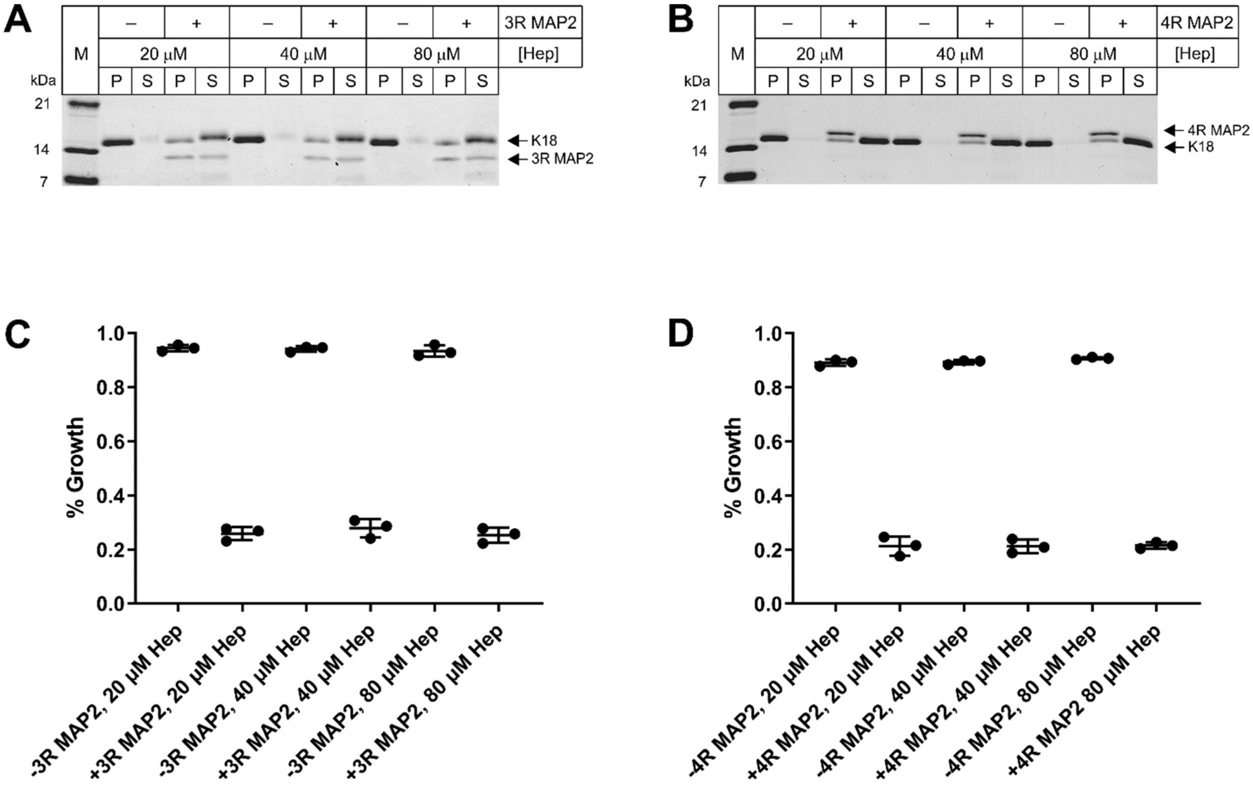


**Figure S4. Heparin titration does not rescue tau aggregation.** **A** and B, K18 seeds (3%) were mixed with K18 monomers (10 μM) in the presence or absence of 5 μM 3R MAP2 (A) or 4 R MAP2 (**B**) and varying concentrations of heparin (20 μM, 40 μM, or 80 μM). The samples were incubated for 16 h at 37 °C, sedimented, and analyzed by SDS-PAGE and Coomassie staining. Representative gels are shown. P, pellet; S, supernatant; M, protein marker; Hep, heparin. **C** and **D**, quantification of fibril growth based on densitometric analysis of gels from three independent experiments. Results for 3R MAP2 inhibition are shown in C, results for 4R MAP2 inhibition in D. From one-way ANOVA, $P < 0.0001$ for each + MAP2 reaction compared to corresponding – MAP2 reaction. Error bars represent means ± S.D.

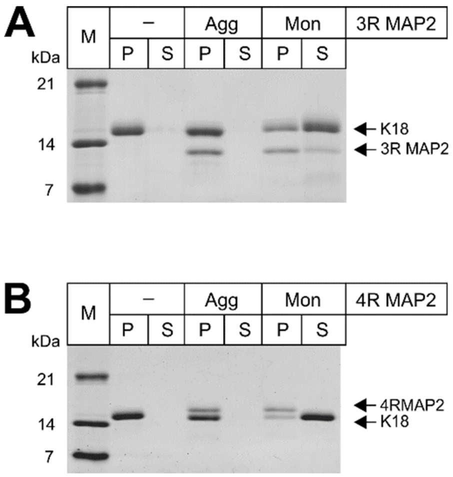


**Figure S5. MAP2 monomers, not aggregates, inhibit tau fibril growth.** 3R MAP2 and 4R MAP2 (5 μM) were incubated for 16 h at 37 °C either in the absence or presence of heparin. Heparin results in the formation of amorphous aggregates, whereas the proteins remain monomeric in its absence. The reactions were subsequently added to K18 monomers (10 μM), mixed with seeds (10%) and heparin (20 μM), and incubated for 6 h at 37 °C. In control experiments MAP2 was absent during fibril growth. The samples were sedimented and analyzed by SDS-PAGE and Coomassie staining. The gels for inhibition experiments with 3R and 4R MAP2 are shown in **A** and **B**, respectively. Agg, amorphous MAP2 aggregates; Mon, MAP2 monomers; P, pellet; S, supernatant.

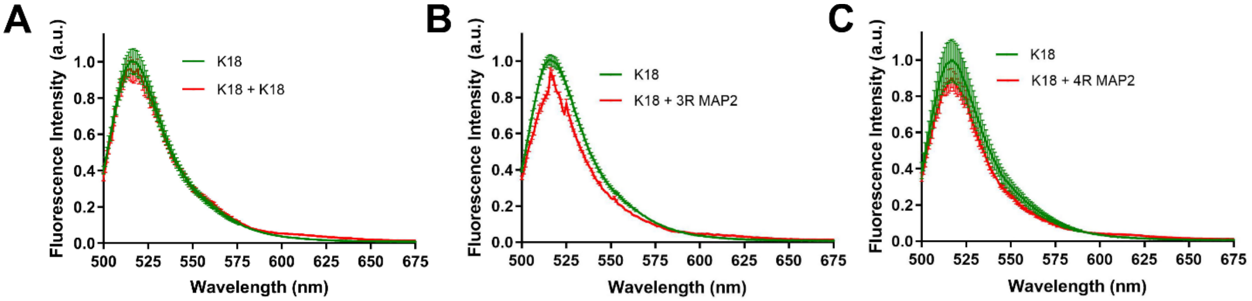


**Figure S6. In the absence of seeds, tau and MAP2 produce no FRET signal.** K18 monomers (1 μM) labeled with Alexa488 (donor fluorophore) were incubated for 1 h at 37 °C. Emission spectra were taken and monomers (1 μM) labeled with Alexa594 (acceptor fluorophore) were added. Added monomers were K18 (**A**), 3R MAP2 (**B**), and 4R MAP2 (**C**). Emission spectra for mixtures of donor- and acceptor-labeled monomers are represented by red traces, emission spectra for donor-labeled K18 by green traces. All experiments were performed in triplicate. Error bars represent means ± S.D. Compared to the seeded reactions shown in Fig. 5, A-C, there are no pronounced peaks visible at 611 nm indicating that in solution, the distances between donor-and acceptor labeled monomers are not within the FRET range.

**A**

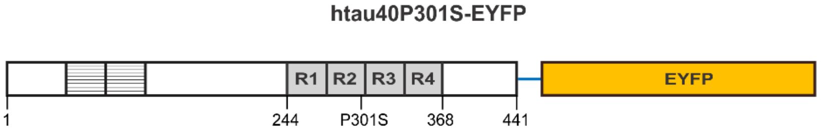


**B**

```
MAEPRQEFEVMEDHAGTYGLGDRKDQGGYTMHQDQEGDTDAGLKESPLQT
PTEDGSEEPGSETSDAKSTPTAEDVTAPLVDEGAPGKQAAAQPHTEIPEG
TTAEEAGIGDTPSLEDEAAGHVTQARMVSKSKDGTGSDDKKAKGADGKTK
IATPRGAAPPGQKGQANATRIPAKTPPAPKTPPSSGEPPKSGDRSGYSSP
GSPGTPGSRSRTPSLPTPPTREPKKVAVVRTPPKSPSSAKSRLQTAPVPM    htau40P301S
PDLKNVKSKIGSTENLKHQPGGGKVQIINKKLDLSNVQSKCGSKDNIKHV
SGGGSVQIVYKPVDLSKVTSKCGSLGNIHHKPGGGQVEVKSEKLDFKDRV
QSKIGSLDNITHVPGGGNKKIETHKLTFRENAKAKTDHGAEIVYKSPVVS
GDTSPRHLSNVSSTGSIDMVDSPQLATLADEVSASLAKQGLGGGGSGGGG    linker
SGGGGSGGGGSMVSKGEELFTGVVPILVELDGDVNGHKFSVSGEGEGDAT
YGKLTLKFICTTGKLPVPWPTLVTTFGYGLQCFARYPDHMKQHDFFKSAM
PEGYVQERTIFFKDDGNYKTRAEVKFEGDTLVNRIELKGIDFKEDGNILG    EYFP
HKLEYNYNSHNVYIMADKQKNGIKVNFKIRHNIEDGSVQLADHYQQNTPI
GDGPVLLPDNHYLSYQSALSKDPNEKRDHMVLLEFVTAAGITLGMDELYK
```

**Figure S7. Mammalian expression construct of tau used in this study. A**, schematic diagram of htau40P301S linked to enhanced yellow fluorescent protein (EYFP). **B**, Amino acid sequence of the expression construct. The htau40P301S sequence (black letters) is connected to EYFP (orange letters) via a 20 amino acid linker (blue) at the C-terminus.

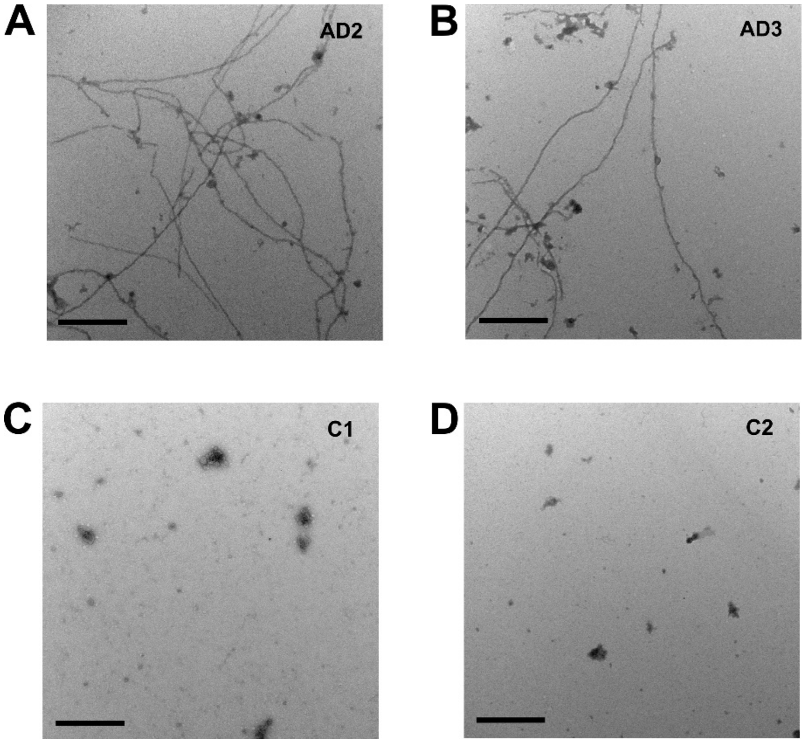


**Figure S8. Tau fibrils can be amplified from AD brain extracts, but not from control extracts. A** and **B**, EM images of amplified htau40 fibrils from AD brain extracts (AD2 and AD3). **C** and **D**, EM images of amplified material from control brain extracts (C1 and C2). The images correspond to the amplification reactions shown in Figure 8. Scale bars, 500 nm.

| Subject | Gender | Age | Postmortem (h) | Neuropathic Dx |
|---|---|---|---|---|
| C1 | M | 74 | N/A | Control |
| C2 | F | 53 | 19.75 | Control |
| AD1 | M | 78 | 5.5 | AD Braak stage VI |
| AD2 | M | 78 | 11.75 | mixed AD/LBD |
| AD3 | M | 81 | 12.5 | AD Braak stage IV/VI |

**Table S1. Brain tissue used in this study.**
